# QSAR Modeling for Multi-Target Drug Discovery: Designing Simultaneous Inhibitors of Proteins in Diverse Pathogenic Parasites

**DOI:** 10.3389/fchem.2021.634663

**Published:** 2021-03-10

**Authors:** Valeria V. Kleandrova, Luciana Scotti, Francisco Jaime Bezerra Mendonça Junior, Eugene Muratov, Marcus T. Scotti, Alejandro Speck-Planche

**Affiliations:** ^1^ Laboratory of Fundamental and Applied Research of Quality and Technology of Food Production, Moscow State University of Food Production, Moscow, Russian Federation; ^2^ Postgraduate Program in Natural and Synthetic Bioactive Products, Federal University of Paraíba, João Pessoa, Brazil; ^3^ Laboratory of Synthesis and Drug Delivery, State University of Paraíba, João Pessoa, Brazil; ^4^ Laboratory for Molecular Modeling, The UNC Eshelman School of Pharmacy, University of North Carolina at Chapel Hill, Chapel Hill, NC, United States

**Keywords:** artificial neural network, fragment, mt-QSAR, multilayer perceptron, multi-target inhibitor, parasites, virtual design, docking

## Abstract

Parasitic diseases remain as unresolved health issues worldwide. While for some parasites the treatments involve drug combinations with serious side effects, for others, chemical therapies are inefficient due to the emergence of drug resistance. This urges the search for novel antiparasitic agents able to act through multiple mechanisms of action. Here, we report the first multi-target model based on quantitative structure-activity relationships and a multilayer perceptron neural network (mt-QSAR-MLP) to virtually design and predict versatile inhibitors of proteins involved in the survival and/or infectivity of different pathogenic parasites. The mt-QSAR-MLP model exhibited high accuracy (>80%) in both training and test sets for the classification/prediction of protein inhibitors. Several fragments were directly extracted from the physicochemical and structural interpretations of the molecular descriptors in the mt-QSAR-MLP model. Such interpretations enabled the generation of four molecules that were predicted as multi-target inhibitors against at least three of the five parasitic proteins reported here with two of the molecules being predicted to inhibit all the proteins. Docking calculations converged with the mt-QSAR-MLP model regarding the multi-target profile of the designed molecules. The designed molecules exhibited drug-like properties, complying with Lipinski’s rule of five, as well as Ghose’s filter and Veber’s guidelines.

## Introduction

Parasitic diseases are dangerous and prevalent health issues, causing high morbidities and mortalities worldwide. Among them, malaria, Chagas’ disease (ChD), African animal trypanosomiasis (AAT), and toxoplasmosis, deserve special attention. From one side, malaria (mainly caused by *Plasmodium falciparum*), although one of the oldest illnesses known by mankind, and yet it remains the deathliest parasitic disease, being responsible for 445,000 deaths and 216 million cases in 2016 (WHO, 2017). On the other hand, we have ChD and AAT, which are the consequences of the infections caused by *Trypanosoma cruzi* and several species belonging to *Trypanosoma* spp. (including *Trypanosoma brucei brucei*), respectively; while ChD continues to threaten millions of people in Mexico, as well as Central and South America (Molyneux et al., 2017; Perez-Molina and Molina, 2018), AAT causes great economic losses due to its devastating mortality on livestock (Giordani et al., 2016; Amisigo et al., 2019). In contrast to malaria, ChD, and AAT, whose negative impacts are located in specific continental areas, toxoplasmosis (caused by *Toxoplasma gondii*) has a worldwide distribution, infecting humans as well as most warm-blooded animals including mammals and birds (Robert-Gangneux and Darde, 2012). In fact, in developed countries such as the United States, toxoplasmosis infects over a million people each year, where this illness is associated with an estimated cost of $3 billion (Aguirre et al., 2019).

In terms of treatment, the parasitic diseases mentioned here present several factors in common that make their eradication a challenge. First, current antiparasitic drugs are associated with many side effects (Forsyth et al., 2016; Grabias and Kumar, 2016; Kwofie et al., 2016; Alday and Doggett, 2017; Buckner et al., 2017; Haeusler et al., 2018). Second, drug resistance has emerged among these parasitic organisms, and consequently, antiparasitic drugs are becoming (or have already become) less effective (Baker et al., 2013; Campos et al., 2017; Montazeri et al., 2018; Conrad and Rosenthal, 2019). Last, as a whole, parasite-parasite interactions are very complex and have been documented and recognized as phenomena that play a crucial role in epidemiology, disease severity, and evolution of parasite virulence (Hellard et al., 2015; Seppala and Jokela, 2016; Dallas et al., 2019; Karvonen et al., 2019).

Screening chemicals through experimental validation is without doubt the most reliable way of identifying antiparasitic agents. However, this trial-and-error approach currently constitutes a time- and cost-ineffective task since the chemical space to be experimentally screened is vast (10^60^ small to medium-sized organic compounds) (Jahnke and Erlanson, 2006). In contrast, computational approaches can accelerate the search for efficacious antiparasitic chemicals, which can later be experimentally validated. At the biomolecular level, many promising computational models and protocols have demonstrated to be essential in early drug discovery, serving as tools for the generation of inhibitors against proteins whose roles are important for the survival or virulence of any of the parasitic species mentioned above. For instance, in the field of malaria research, recent works have focused on the application of an integrative multi-kinase approach (Lima et al., 2019), the identification of malarial allosteric modulators combining molecular dynamics simulations and dynamic residue network analysis (Amusengeri et al., 2019), the ensemble of ligand-based computational models for virtual screening of falcipain-2 inhibitors (Alberca et al., 2019), and quantitative structure-activity relationships (QSAR) for the study of N-myristoyltransferase inhibitors (Santos-Garcia et al., 2018). Regarding ChD, a wide range of *in silico* approaches have been reported to discover protein inhibitors with a special focus on cruzipain (Palos et al., 2017; Dos Santos et al., 2018; Herrera-Mayorga et al., 2019). Following with AAT, several works have reported the use of computational tools to accelerate the search for inhibitors of different targets (Latorre et al., 2016; Di Pisa et al., 2017; Kimuda et al., 2019; Zacharova et al., 2019). Finally, the importance of computer-aided drug discovery has also been evidenced in the identification of different protein inhibitors to tackle toxoplasmosis (Welsch et al., 2016; Rosada et al., 2019; Zhang et al., 2019).

However, despite the growing influence of the computational methods in antiparasitic research, at least one of the three following drawbacks remains. First, computational models use relatively small datasets of structurally related molecules. Second, they lack sufficiently clear physicochemical and/or structural information to guide the design of new and potent protein inhibitors. Last, computational models have been based on only one therapeutic target/protein. All this urges the development of advanced computational models, suggesting that the efforts of the scientific community to speed up the eradication of diseases caused by the aforementioned parasites should focus on the multi-target drug discovery paradigm (Ravikumar and Aittokallio, 2018). In this context, several research groups have emphasized the development of a series of multi-target QSAR (mt-QSAR) models to perform virtual screening of molecules at both biomolecular- and microorganism-based levels (Prado-Prado et al., 2008; Prado-Prado et al., 2010a; Prado-Prado et al., 2010b; Garcia et al., 2011). Yet, no mechanistic, physicochemical, or structural interpretations have been reported for these models.

Currently, there is no computational approach capable of designing and predicting multi-target inhibitors of proteins present in different parasitic species. An *in silico* tool with such capabilities could take advantage of the fact that many parasitic proteins/targets identified to date are conserved across parasitic species (Cowell and Winzeler, 2019); a multi-target computational model would be of great value in both filtering the chemical space in the search for versatile inhibitors against diverse parasitic proteins and guiding the fast and accurate generation of new and potent antiparasitic agents able to act through different mechanisms of action.

Considering all the aforementioned ideas, we introduce here the first mt-QSAR model based on multilayer perceptron network (mt-QSAR-MLP), providing the theoretical foundations for the prediction of chemicals with potential multi-target activity against five parasitic proteins, namely plasmepsin 2 and dihydroorotate dehydrogenase (*P. falciparum*), as well as cruzipain (*T. cruzi*), dihydrofolate reductase (*T. gondii*), and glycylpeptide N-tetradecanoyltransferase (*T. brucei brucei*). Also, we computationally demonstrate that a series of newly designed molecules are worth synthesizing in the future by considering a combination of four factors: 1) they were rationally designed by assembling different molecular fragments according to the physicochemical and structural interpretation of the mt-QSAR-MLP model, 2) they were predicted by the mt-QSAR-MLP as potent multi-target inhibitors of the parasitic proteins, 3) the results of the docking calculations also converges with the predictions from the mt-QSAR-MLP model regarding the multi-target profile of the designed molecules, and 4) the designed molecules were estimated to have good synthetic accessibility.

## Materials and Methods

### Database and Calculation of the Molecular Descriptors

The chemical and biological data were extracted from ChEBML (Gaulton et al., 2012) and contained information regarding the inhibitory potency, i.e., the concentration required to cause 50% inhibition (IC_50_) in any of the five parasitic proteins mentioned above. The dataset was curated in terms of removing all the molecules with missing features such as SMILES, values, units of activity, and duplicates. The present dataset was formed by 2,249 different molecules, and each of them was experimentally tested against only one parasitic protein. In the dataset each molecule was classified as active [*IA*_*i*_(*tg*) = 1] or inactive [*IA*_*i*_(*tg*) = −1], with *IA*_*i*_(*tg*) being a binary variable that indicated the inhibitory activity of *ith* molecule against a defined target/protein. Thus, a molecule was annotated as active if IC_50_ ≤ 800 nM for Plasmepsin 2 (*P. falciparum*), IC_50_ ≤ 820 nM for dihydroorotate dehydrogenase (*P. falciparum*), IC_50_ ≤ 890 nM for cruzipain (*T. cruzi*), IC_50_ ≤ 250 nM for dihydrofolate reductase (*T. gondii*), or IC_50_ ≤ 270 nM for glycylpeptide N-tetradecanoyltransferase (*T. brucei brucei*). In any other case, the molecules were considered inactive. It should be pointed out that the cutoff values selected in this study comply with two important aspects. From one side, by being in the submicromolar range, they ensure the rigorous search for potent hits, a process which, in most drug discovery campaigns usually starts at the micromolar range (Anderson, 2003). On the other hand, in general terms, these cutoff values prevent any excessive imbalance between the number of molecules assigned as active and those labeled as inactive. Finally, the selected cutoffs maintain the number of molecules annotated as active as high as possible; this increases the chemical diversity, which is required when using the mt-QSAR-MLP model to rationally design new molecules. Notice that if a unified cutoff value of the inhibitory activity is selected, then, at least one of two situations will happen: 1) data involving on or more of the parasitic proteins will be considerably imbalanced (reduced chemical diversity among active molecules) which is detrimental to the predictive power of any model, or 2) even if a unified cutoff is set, it will remarkably decrease the rigor of the mt-QSAR-MLP model to search for (and/or design) potent and versatile inhibitors against several parasitic proteins.

The SMILES codes of all the molecules reported in the dataset were stored in a file of type *.smi. This file was converted to *.sdf using the program Standardizer v19.18.0 (ChemAxon, 1998–2019). During the conversion process, as the purpose was to obtain the connectivity table for each molecule, no standardization actions were applied. Following, the computer program QuBiLS-MAS v1.0 (Valdés-Martini et al., 2012; Valdes-Martini et al., 2017) used the file *.sdf as the input for the calculation of the molecular descriptors known as total and local atom-based quadratic indices. When doing so, QuBiLS-MAS v1.0 performed these calculations by considering theoretical aspects such as the algebraic form (quadratic), constrains (atom-based), matrix form (mutual probability). The quadratic indices mentioned here considered all the elements of the mutual probability matrix, and they used the Manhattan distance as the aggregator operator. The reason to select quadratic indices is based on their wide applicability as reported in several works focused on computer-aided drug discovery (Marrero-Ponce et al., 2011; Medina Marrero et al., 2015; Speck-Planche et al., 2015). The quadratic indices can be calculated according to the following mathematical formalism:

$$TmpAq_{\text{k}}\left(x\right)=\overset{n}{\underset{i=1}{\sum }}\overset{n}{\underset{j=1}{\sum }}{}^{\text{k}}mp_{ij}.x_{i}.x_{j}$$ (1)

$$LmpAq_{k}\left(x\right)Z=\overset{n}{\underset{i=1}{\sum }}\overset{n}{\underset{j=1}{\sum }}{}^{\text{k}}mp_{ijZ}.x_{i}.x_{j}$$ (2)

In Eqs. 1, 2, *TmpAq*_k_(*x*) and *LmpAq*_k_(*x*)*Z* represent the total and local atom-based quadratic indices of the mutual probability matrix, respectively. The symbol *x* refers to any atomic physicochemical property such as hydrophobicity (*HYD*), electronegativity (*E*), atomic weight (*AW*), polarizability (*POL*), polar surface area (*PSA*), or volume (V). It should be pointed out that while in Eq. 1 ${}^{\text{k}}mp_{ij}$ expresses the adjacency between any two atoms in a molecule, in Eq. 2, ${}^{\text{k}}mp_{ijZ}$ has a similar meaning. Nevertheless, ${}^{\text{k}}mp_{ijZ}$ depends on specific atoms types (*Z*) such as hydrogen bond acceptors, aliphatic and aromatic carbons, methyl groups, halogens, and heteroatoms. Both *TmpAq*_k_(*x*) and *LmpAq*_k_(*x*)*Z* describe a defined atom *i* and its chemical environment (formed by the *jth* neighbors) at the topological distance *d* = *k*.

The purpose here is to develop an mt-QSAR-MLP model as a computational tool able to predict inhibitory activity against dissimilar proteins present in diverse parasites. Thus, although the molecular descriptors calculated in Eqs. 1, 2 can characterize the chemical structure of the molecules, they will not be able to discriminate the structural and physicochemical information present in a molecule when this is tested against more than one target/protein. In this context, several works have applied an adaptation of the Box-Jenkins approach (used in time series analysis) to calculate multi-target molecular descriptors in a two-steps manner (Marzaro et al., 2011; Speck-Planche and Kleandrova, 2012a; Speck-Planche et al., 2012; Alonso et al., 2013; Speck-Planche et al., 2013; Romero Duran et al., 2014; Romero-Duran et al., 2016):

$$avgQI\left(tg\right)=\frac{1}{n\left(tg\right)}\times \overset{n\left(tg\right)}{\underset{a=1}{\sum }}QI_{a}$$ (3)

In Eq. 3, *QI*_*a*_ is any of the quadratic indices mentioned above. The symbol *avgQI*(*tg*) represents the average of any quadratic index for all the molecules in the training set labeled as active and tested against the same parasite protein. Consequently, *n*(*tg*) denotes the number of active molecules/cases (also present in the training set) that were assayed against the same protein. The second step applies the following formula:

$$DQI_{a}\left(tg\right)=\frac{QI_{a}-avgQI\left(tg\right)}{\left(QI_{MX}-QI_{MN}\right)\times \sqrt{p\left(tg\right)}}$$ (4)

In Eq. 4, *DQI*_*a*_(*tg*) is a multi-target descriptor and depends on the chemical structure of a molecule and the parasite protein against which that molecule was tested; this descriptor measures how much any molecule structurally deviates from a group of molecules assigned as active and assayed against the same protein. On the other hand, *QI*_*MX*_ and *QI*_*MN*_ are the maximum and minimum values of each quadratic index (in the training set), respectively. Last, *p*(*tg*) is the *a priori* probability of finding a compound tested against a specific parasite protein; it is calculated as the ratio of the number of molecules in the training set assayed against a given protein to the total number of compounds present in the training set.

### Building the Mt-QSAR-MLP Model

**FIGURE 1:**
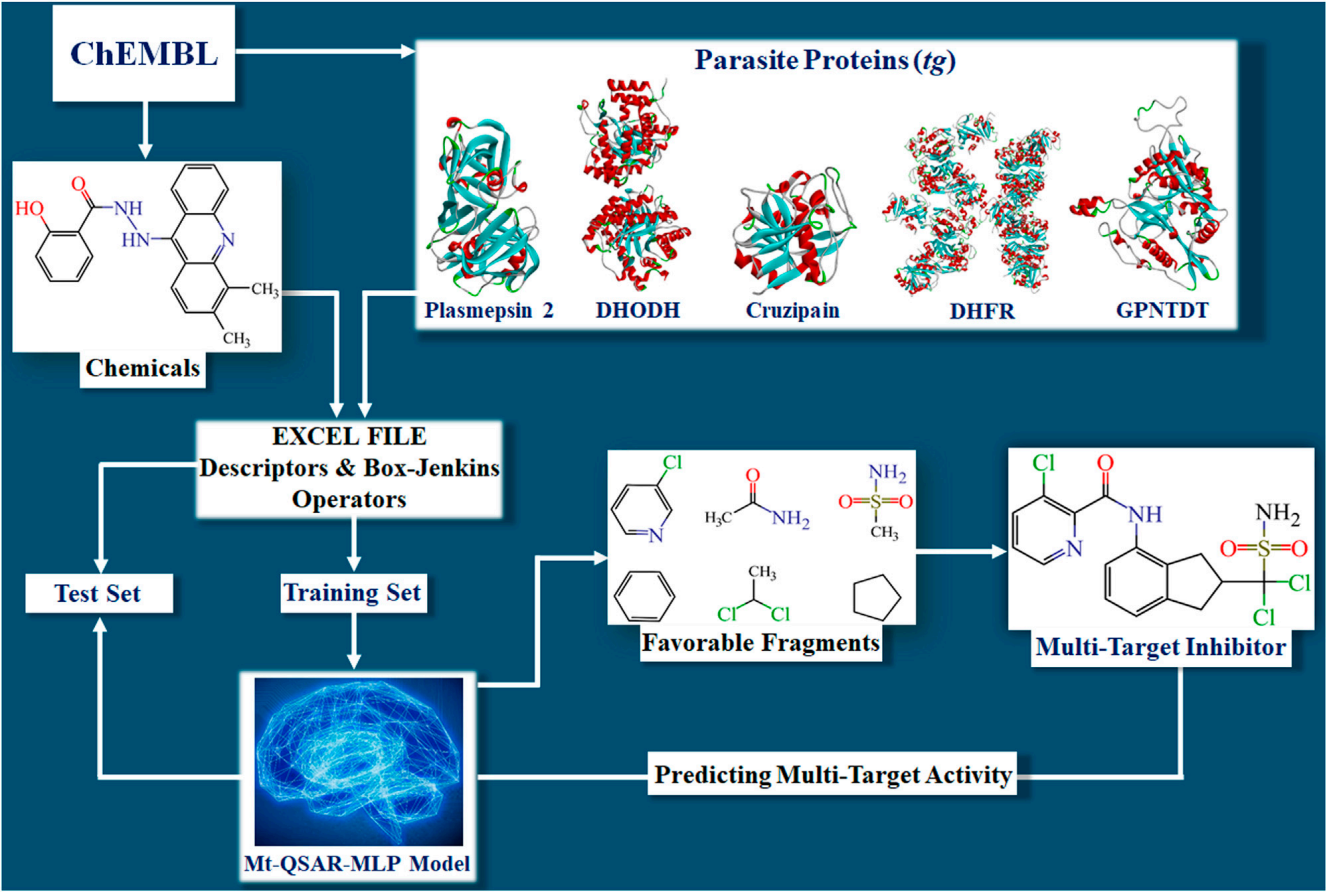
Steps involved in the construction of the mt‐QSAR‐MLP model. The abbreviations DHODH, DHFR, and GPNTDT refer to the parasite proteins named dihydroorotate dehydrogenase, dihydrofolate reductase, and glycylpeptide N-tetradecanoyltransferase, respectively.

Developing the mt-QSAR-MLP occurred in different steps (Figure 1). First, the dataset containing the 2,249 molecules was split into training and test sets according to the following procedure. For each parasitic protein, the molecules were sorted according to their increasing IC_50_ values. Then, for each protein, the first three molecules were assigned to the training set while the fourth molecule was assigned to the test set. Such a ratio of 3:1 was repeated in the whole dataset. Thus, the training set was employed to search for the best model and was formed by 1,691 molecules (75.19%), 788 considered as active and 903 annotated as inactive. The test set was meant to demonstrate the predictive power of the mt-QSAR-MLP model; this set contained 558 molecules (remaining 24.81% of the dataset), 259 assigned as active and 299 considered as inactive.

Second, it is known that the random forest (RF) is one of the most popular machine learning methods to obtain predictive models (Hastie et al., 2009). In this work, RF was used as a variable selection strategy. In this sense, and using the descriptors of the type *DQI*_*a*_(*tg*) as inputs, the RF package of the computer program software STATISTICA v13.5.0.17 (TIBCO-Software-Inc., 2018) was employed to perform multiple runs to find the best mt-QSAR-RF model. In doing so, we used default values for the different parameters in the RF algorithm [number of predictors: 259; number of trees: 100; subsample proportion: 0.5; seed for random number generator: 1; minimum number of cases: 56; minimum number in child node: 5; maximum number of levels: 10; maximum number of nodes: 100; cycles to calculate mean error: 10; percentage decrease in training error: 5]. While selecting the most influential descriptors (highest importance values) in the mt-QSAR-RF model, we conducted a correlation analysis for the molecular descriptors of the type *DQI*_*a*_(*tg*) by computing the Pearson’s correlation coefficient (*PCC*) (Pearson, 1895); only the descriptors having pairwise correlation values in the interval −0.7 < *PCC* < 0.7 were chosen.

Artificial neural networks (ANNs) was used as the data analysis method to search for the best model, the architecture known as the multi-layer perceptron (MLP) were examined because of its popularity, accuracy, and relative ease of convergence. When training the MLP networks, we employed the Broyden-Fletcher-Goldfarb-Shanno (BFGS) algorithm, setting the number of epochs to be 300. To obtain the most appropriate mt-QSAR-MLP model, several runs were performed using the ANNs package of STATISTICA v13.5.0.17 (TIBCO-Software-Inc., 2018) while inspecting the statistical indices known as accuracy [*Ac*(%)] and Matthews’ correlation coefficient (*MCC*) (Matthews, 1975), as well as sensitivity [Sn(%)] and specificity [*Sp*(%)] and their local counterparts [Sn(%)]*tg*, and [*Sp*(%)]*tg*. It should be highlighted that while [Sn(%)] and [*Sp*(%)] give an idea of the global statistical quality (training set) and predictive power (test set) of the mt-QSAR-MLP model [Sn(%)]*tg*, and [*Sp*(%)]*tg* provide similar information but depending on each of the five proteins reported in this work. Only the mt-QSAR-MLP model exhibiting the highest values of [Sn(%)] [*Sp*(%)] [Sn(%)]*tg*, and [*Sp*(%)]*tg* was selected.

### Molecular Docking

When performing docking calculations, we used the software Molegro Virtual Docker v6.0.1 (Thomsen and Christensen, 2006), employing the same protocol as recently reported in (Speck-Planche and Scotti, 2019). We retrieved all the crystallographic structures from the Protein Data Bank (PDB) (Berman et al., 2000). In doing so, we considered the PDB IDs 2BJU (Prade et al., 2005), 6I55 (Pippione et al., 2019), 1ME3 (Huang et al., 2003), and 4KY4 (Zaware et al., 2013) for the proteins plasmepsin 2 (*P. falciparum*), dihydroorotate dehydrogenase (*P. falciparum*), cruzipain (*T. cruzi*), and dihydrofolate reductase (*T. gondii*), respectively. These PDB files contained the aforementioned proteins complexed with their corresponding reference ligands. We validated the docking protocol by redocking each reference ligand into the active site of the protein for which the corresponding complex with that protein was experimentally reported. In the case of the protein glycylpeptide N-tetradecanoyltransferase (*T. brucei brucei*), no crystallographic structure has been reported to date. Therefore, we relied on homology modeling to create the 3D-structure of this protein. In this sense, we employed SWISS-MODEL (Waterhouse et al., 2018), which is fully automated protein homology modeling webserver. When performing homology modeling with SWISS-MODEL, we entered the UniprotID Q388H8, which corresponded to glycylpeptide N-tetradecanoyltransferase (*T. brucei brucei*). Then, SWISS-MODEL performed an automatic search for different proteins’ amino acid sequences to use them as templates, selecting the most reliable model of the 3D-structure of glycylpeptide N-tetradecanoyltransferase (*T. brucei brucei*). Last all the interactions for each ligand-protein complex were visualized by the Discovery Studio Visualizer v19.1 (BIOVIA, 2018).

## Results and Discussion

### The Mt-QSAR-MLP Model

The best mt-QSAR-MLP model found by us had the profile MLP 9–27–2, which means that nine nodes [molecular descriptors of the type *DQI*_*a*_(*tg*)] were used in the input layer, 27 nodes in the hidden layer with a logistic activation function, while in the output layer (based on a softmax function), the number two refers to the two possible categorical values (−1 and 1) of the variable of predicted inhibitory potency [*Pred*_*IA*_*i*_(*tg*)]. Details regarding the different molecular descriptors used to build the mt-QSAR-MLP model appear in Table 1. At the same time, all the chemical and biological data can be gathered from Supplementary Material S1.

**TABLE 1:** Molecular descriptors and their definitions.

Molecular descriptor^a^	Definition
***D*[*TmpAq*2(*HYD*)]*tg***	Deviation of the total atom-based quadratic index (order 2) of the mutual probability matrix weighted by the *HYD*.
***D*[*TmpAq*5(*PSA*)]*tg***	Deviation of the total atom-based quadratic index (order 5) of the mutual probability matrix weighted by the *PSA*.
***D*[*LmpAq*6(*POL*)*A*]*tg***	Deviation of the local atom-based quadratic index (order 6) of the mutual probability matrix weighted by the *POL*. This descriptor considers only the hydrogen bond acceptors and their neighbor atoms
***D*[*LmpAq*0(*PSA*)*A*]*tg***	Deviation of the local atom-based quadratic index (order 0) of the mutual probability matrix weighted by the *PSA*. This descriptor considers only the presence of hydrogen bond acceptors
***D*[*LmpAq*6(*PSA*)*A*]*tg***	Deviation of the local atom-based quadratic index (order 6) of the mutual probability matrix weighted by the *PSA*. This descriptor considers only the hydrogen bond acceptors and their neighbor atoms
***D*[*LmpAq*6(*POL*)*C*]*tg***	Deviation of the local atom-based quadratic index (order 6) of the mutual probability matrix weighted by the *POL*. This descriptor considers only the aliphatic carbons and their neighbor atoms
***D*[*LmpAq*1(*V*)*C*]*tg***	Deviation of the local atom-based quadratic index (order 1) of the mutual probability matrix weighted by the atomic *V*. This descriptor considers only the aliphatic carbons and their adjacent atoms
***D*[*LmpAq*5(*POL*)*P*]*tg***	Deviation of the local atom-based quadratic index (order 5) of the mutual probability matrix weighted by the *POL*. This descriptor considers only the aromatic carbons and their neighbor atoms
***D*[*LmpAq*0(*PSA*)*Y*]*tg***	Deviation of the local atom-based quadratic index (order 0) of the mutual probability matrix weighted by the *PSA*. This descriptor considers only the presence of heteroatoms

^a^ All these molecular descriptors depend on both the chemical structure of a molecule and the target/protein against which the molecule was experimentally assayed.

In terms of statistical quality, the mt-QSAR-MLP model exhibited *Ac*(%) = 84.68%, indicating that 1,432 out of 1,691 molecules were correctly classified. In the test set, also a good performance was achieved; 444 out of 558 molecules were correctly predicted [*Ac*(%) = 79.57%]. In addition to the values of accuracy mentioned here, relatively high values for the other statistical indices were obtained (Table 2). For instance, Sn(%) and *Sp*(%) had values higher than 83% in the training set, while for the test set, they exhibited values around 80%. Simultaneously, *MCC* took values higher than 0.59, and given their closeness to one (perfect performance) more than to zero (for a random predictor), it can be inferred that there is a strong correlation between the observed [*IA*_*i*_(*tg*)] and predicted [*Pred*_*IA*_*i*_(*tg*)] values of the inhibitory activity. For each molecule in the dataset information regarding its classification performed by the mt-QSAR-MLP model is reported in Supplementary Material S1.

**TABLE 2:** Internal quality and predictive performance of the mt-QSAR-MLP model.

SYMBOLS^a^	Training set	Test set
***N***_**Active**_	788	259
***CC***_**Active**_	676	208
***Sn*(%)**	85.79%	80.31%
***N***_**Inactive**_	903	299
***CC***_**Inactive**_	756	236
***Sp*(%)**	83.72%	78.93%
***MCC***	0.694	0.591

^a^ ***N***_**Active**_–Number of molecules annotated as active; ***N***_**Inactive**_–Number of molecules annotated as inactive; ***CC***_**Active**_–Molecules correctly classified as active; ***CC***_**Inactive**_–Molecules correctly classified as inactive; ***Sn*(%)**—Sensitivity (percentage of molecules correctly classified as active); ***Sp*(%)**—Specificity (percentage of molecules correctly classified as inactive); ***MCC***–Matthews’ correlation coefficient.

Although any predictive model should have relatively high values of as Sn(%) and *Sp*(%), for the case of an mt-QSAR model, it is also important that the local sensitivities [Sn(%)]*tg* and specificities [*Sp*(%)]*tg* for each protein should also exhibit values as high as possible. In this context [Sn(%)]*tg* and [*Sp*(%)]*tg* were higher than 80% in the training set whereas, for the test set, values higher than 72% and 75% were computed for these two statistical indices, respectively. Details of the different [Sn(%)]*tg* and [*Sp*(%)]*tg* values are available in Supplementary Material S2. The only exception was[*Sp*(%)]*tg* in the case of the protein dihydrofolate reductase (*T. gondii*) for which values of 69.92% and 65.82% for training and test sets, respectively. We attribute the wrong predictions to the fact that the molecular descriptors *DQI*_*a*_(*tg*) are not capable of considering all the differences in the chemical structures of the molecules which produce the corresponding changes in their inhibitory potency against the parasite proteins. This is another confirmation that the ability of the molecular descriptors reported to date to contain information on the complexity and diversity of the molecules is limited (Todeschini and Consonni, 2009). In any case, the joint analysis of the global statistical indices and the local sensitivities and specificities demonstrate the good statistical quality and predictive power of the mt-QSAR-MLP model.

### Applicability Domain

The assessment of the applicability domain (AD) of the mt-QSAR-MLP model was carried out by employing a modification of the descriptor space approach (Sahigara et al., 2012), which establishes that the maximum and minimum values of each molecular descriptor (in the training set) are the boundaries of the AD of a model. Here, we defined the maximum and minimum values of each *DQI*_*a*_(*tg*) descriptor in the mt-QSAR-MLP by considering only those molecules in the training set that were correctly classified (Speck-Planche, 2018). For each molecule present in the dataset, a local score of applicability domain for each of its *DQI*_*a*_(*tg*) descriptors was assigned. In this sense, if for a molecule, a given descriptor value was within the interval defined by the maximum and minimum values, the local score was equal to one; otherwise, the local score was equal to zero. This procedure was repeated for each *DQI*_*a*_(*tg*) descriptor in the mt-QSAR-MLP model. In the end, the sum of all the scores for each molecule was calculated, yielding the total score of the applicability domain (TSAD). Thus, as the mt-QSAR-MLP model was built from nine molecular descriptors, only the molecules with TSAD = 9 were considered to be within the AD (Supplementary Material S3).

### Molecular Descriptors and Their Physicochemical and Structural Meanings

Interpreting any QSAR model is crucial for the understanding of the physicochemical properties and structural features that govern the enhancement (or the diminution) of the biological activity under study. To provide a more complete interpretation of the mt-QSAR-MLP model developed in this work, we have combined chemical reasoning, statistical aspects, a fragment-based analysis into a single explanation.

Chemical reasoning focuses on the fact that the *DQI*_*a*_(*tg*) descriptors employed to build the mt-QSAR-MLP model are characterized by two important elements. First, the topological distance *d* = *k* [with *k* being the order of each *DQI*_*a*_(*tg*)] expresses the number of bonds (without considering bond multiplicity) that exist between any two atoms in a molecule. Chemically speaking, by using this information, it is possible to know the regions in a molecule where atoms exhibiting certain physicochemical properties can be placed with respect to their neighbor atoms. Second, the *DQI*_*a*_(*tg*) descriptors also cover lower topological distances. For instance, a *DQI*_*a*_(*tg*) descriptor of order six will describe information at the topological distance equal to six but also at the topological distances of two and three. This is because *DQI*_*a*_(*tg*) descriptors also measure the degree of concentration of a physicochemical property at the topological distance *d* ≤ *k*. Chemical reasoning will provide information in terms of the distributions of the atoms with different physicochemical properties throughout the entire structure of a molecule.

**FIGURE 2:**
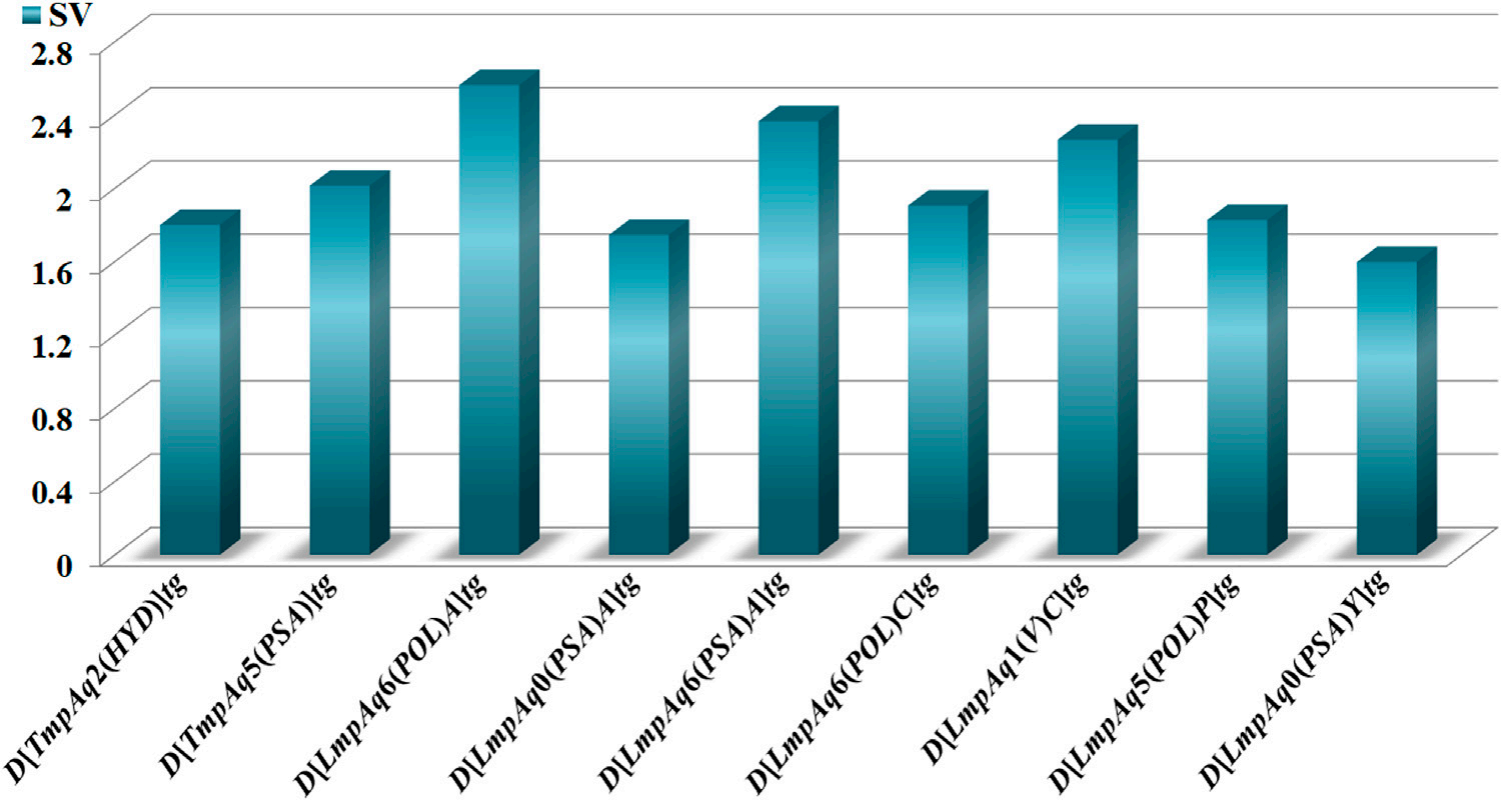
Molecular descriptors and their statistical influences in the mt-QSAR-MLP model.

The statistical aspects focused on two elements, the relative importance of each quadratic index in the mt-QSAR-MLP. Such information was provided by carrying out a sensitivity analysis with the ANN package of STATISTICA v13.5.0.17. This permitted us to estimate the sensitivity values of the *DQI*_*a*_(*tg*) descriptors; the highest *SVs* corresponded to those which were the most influential in the mt-QSAR-MLP model (Figure 2). The other statistical element is the tendency of variation of the *DQI*_*a*_(*tg*) descriptors. We would like to emphasize that the model developed in this work is non-linear. Consequently, there is no equation from which the variation (increase or diminution) in the values of each *DQI*_*a*_(*tg*) descriptor can be determined. To solve this inconvenience, we applied the approach reported by Speck-Planche and co-workers (Speck-Planche and Kleandrova, 2012b; Speck-Planche, 2018; Speck-Planche, 2019). Basically, for each *DQI*_*a*_(*tg*) descriptor present in the mt-QSAR-MLP model, two average values were calculated: one for the molecules annotated as active and the other for the molecules assigned as inactive. It is important to highlight that the calculation of the two averages of each *DQI*_*a*_(*tg*) descriptor was carried out by considering only those molecules in the training set that were correctly classified by the mt-QSAR-MLP model. Comparing the two average values between each other offers the possibility of knowing how the value of a given *DQI*_*a*_(*tg*) descriptor should vary to enhance the biological effect under study, in this case, the multi-target activity against different parasite proteins. The class-based averages and the corresponding tendency of variation for each *DQI*_*a*_(*tg*) descriptor are reported in Table 3.

**TABLE 3:** Tendencies of variation of the molecular descriptors in the mt-QSAR-MLP model according to the classes-based means’ approach.

Symbol	Active	Inactive	Tendency^a^
***D*[*TmpAq*2(*HYD*)]*tg***	7.92 × 10^–3^	−1.14 × 10^–1^	Increase
***D*[*TmpAq*5(*PSA*)]*tg***	1.82 × 10^–3^	3 × 10^–2^	Decrease
***D*[*LmpAq*6(*POL*)*A*]*tg***	4.39 × 10^–3^	−6.38 × 10^–3^	Increase
***D*[*LmpAq*0(*PSA*)*A*]*tg***	−3.43 × 10^–3^	9.63 × 10^–2^	Decrease
***D*[*LmpAq*6(*PSA*)*A*]*tg***	3.09 × 10^–3^	−5.63 × 10^–3^	Increase
***D*[*LmpAq*6(*POL*)*C*]*tg***	−7.22 × 10^–4^	2.93 × 10^–2^	Decrease
***D*[*LmpAq*1(*V*)*C*]*tg***	2.32 × 10^–3^	−5.77 × 10^–2^	Increase
***D*[*LmpAq*5(*POL*)*P*]*tg***	4.84 × 10^–3^	−4.53 × 10^–2^	Increase
***D*[*LmpAq*0(*PSA*)*Y*]*tg***	6.14 × 10^–4^	5.26 × 10^–2^	Decrease

^**a**^ Tendency–It indicates the type of variation (increase or diminution) of a descriptor in order to enhance the multi-target activity against the different parasite proteins.

Regarding the fragment-based analysis, there is solid evidence that demonstrates that any topological (graph-based) descriptor calculated for a molecule can be expressed as the number of times in which different fragments (both connected and disconnected) appear in that molecule (Baskin et al., 1995). This means that the information content of any topological descriptor can be associated with a series of fragments. From a substructural point of view, the *DQI*_*a*_(*tg*) descriptors present in the mt-QSAR-MLP model constitute a class of topological descriptors, and therefore, while interpreting them, different fragments whose presence leads to favorable variations (responsible for increasing the inhibitory activity) of these *DQI*_*a*_(*tg*) descriptors can be extracted (Speck-Planche, 2019; Kleandrova and Speck-Planche, 2020).

**FIGURE 3:**
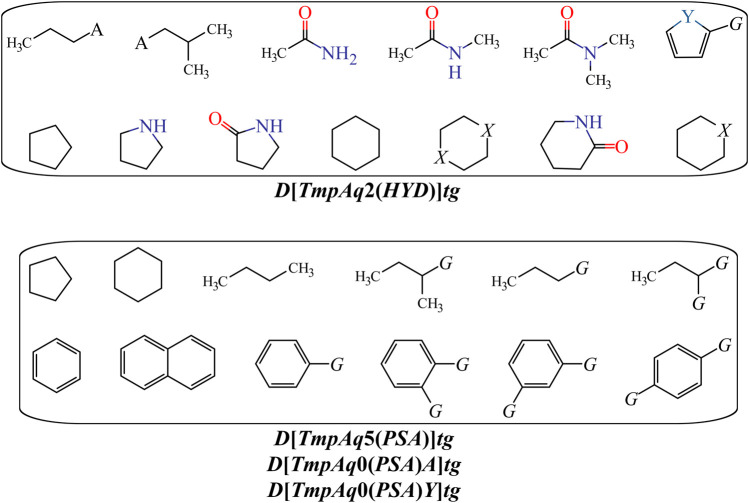
Fragments with positive influence to the increase of the hydrophobic contribution {***D*[*TmpAq2*(*HYD*)]*tg***} or the decrease of the PSA {***D*[*TmpAq5*(*PSA*)]*tg***, ***D*[*LmpAq0*(*PSA*)*A*]*tg***, and ***D*[*LmpAq0*(*PSA*)*Y*]*tg***}. Here, A = −NH2, −OH, or R (alkyl group); X = O or −NH−; Y = S; G = Cl, Br, or I.

We have ***D*[*TmpAq*2(*HYD*)]*tg*** (the seventh most influential descriptor), which expresses the augmentation of the joint hydrophobic contribution (multiplication of the atomic hydrophobicity) of any two atoms placed at the topological distance of two. We would like to highlight that the atomic hydrophobicities used in this work are based on the hydrophobicity scale proposed by Ghose and co-workers (Ghose et al., 1998). According to this scale, aliphatic carbon atoms will have negative hydrophobicity values except for those of the type CHX3, CR2X2, CRX3, and CX4 (X is an electronegative atom such as O, N, S, P, Se, or any halogen). Nitrogen and oxygen atoms have also been reported to have negative hydrophobicity values; exceptions are pyrrolic nitrogen (or furan oxygen) atoms, nitrogen from amines (or oxygen from ethers) having attached two aromatic (or heteroaromatic) rings, and all the tertiary amines. That being said, it is clear that the presence of aliphatic amines and ethers, regardless of whether they are acyclic or cyclic) have favorable contributions to the increase of ***D*[*TmpAq*2(*HYD*)]*tg***. A non-exhaustive but useful list of suitable generic fragments is depicted in Figure 3.

In Table 3 and Figure 3, we can see that the diminution of the *PSA* is governed by the descriptors ***D*[*TmpAq*5(*PSA*)]*tg***, ***D*[*LmpAq*0(*PSA*)*A*]*tg***, and ***D*[*LmpAq*0(*PSA*)*Y*]*tg***, which are rank fourth, eighth, and ninth among the most significant descriptors, respectively. Particularly, ***D*[*TmpAq*5(*PSA*)]*tg*** considers the decrease of the *PSA* of any two atoms placed at the topological distance of five while ***D*[*LmpAq*0(*PSA*)*A*]*tg*** and ***D*[*LmpAq*0(*PSA*)*Y*]*tg*** indicate the reduction of the *PSA* depending on hydrogen bond acceptors and heteroatoms, respectively. Altogether, these three descriptors express that fragments containing aromatic rings (both unsubstituted and substituted) as well as aliphatic rings and chains are desirable for the favorable decrease of the *PSA*. An interesting fact is that in most of the molecules, the *PSA* strongly depends on the presence of nitrogen and oxygen atoms, which is characterized by both descriptors ***D*[*LmpAq*0(*PSA*)*A*]*tg*** and ***D*[*LmpAq*0(*PSA*)*Y*]*tg***. Therefore, these two descriptors should correlate with each other. This, however, doesn’t happen because ***D*[*LmpAq*0(*PSA*)*Y*]*tg*** also considers other atoms with *PSA* such as sulfur and phosphorus. In the database used to build the mt-QSAR-MLP model, there are many compounds with different functional groups containing sulfur, which is the main factor preventing the existence of a correlation between ***D*[*LmpAq*0(*PSA*)*A*]*tg*** and ***D*[*LmpAq*0(*PSA*)*Y*]*tg***. In the end, the number of atoms with values of *PSA* different from zero should be kept as low as possible.

**FIGURE 4:**
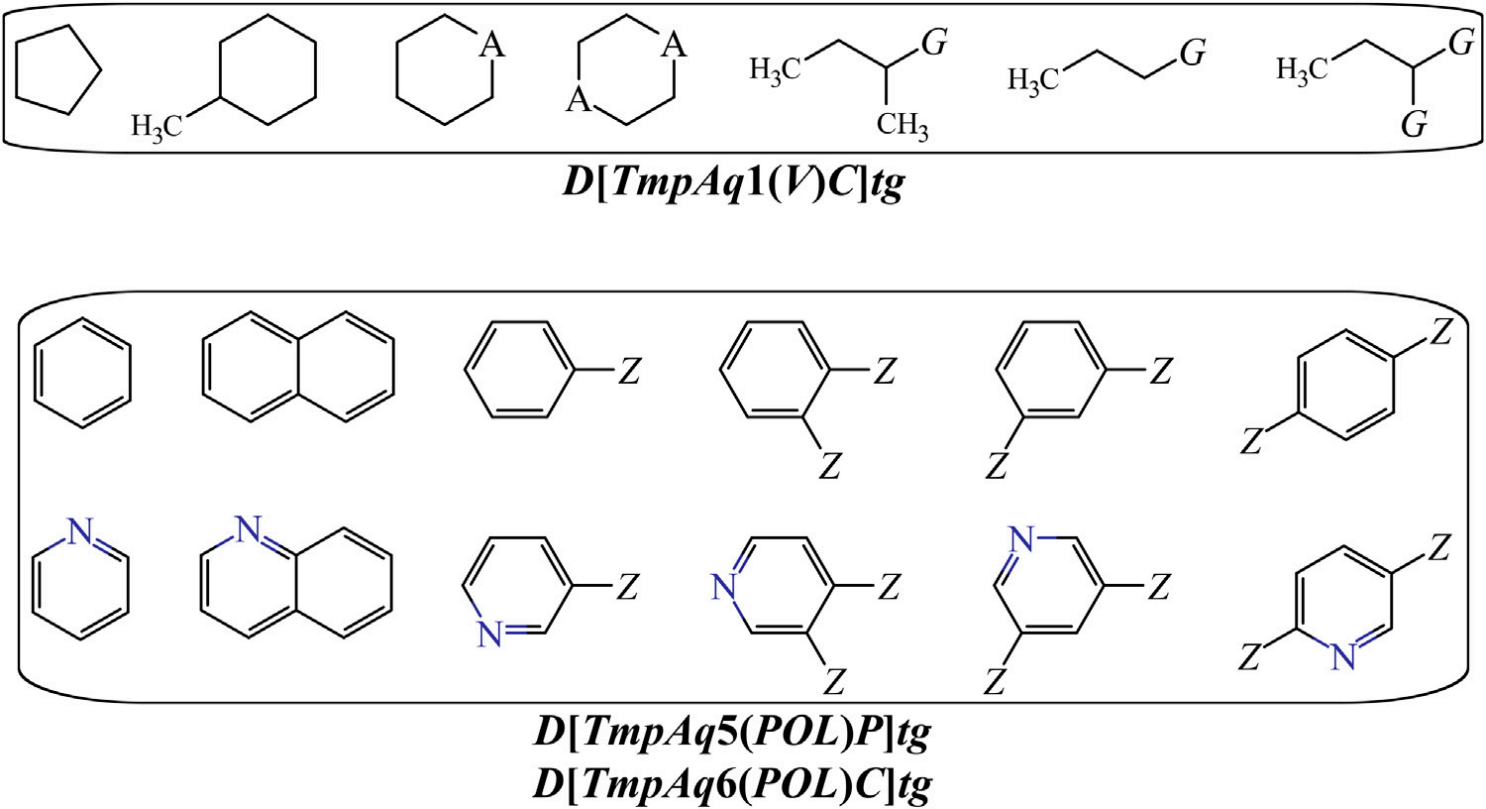
Substructures exhibiting with positive contributions to the desirable increase of the *V* {***D*[*LmpAq1*(*V*)*C*]*tg***} or the favorable variation of the POL {***D*[*LmpAq5*(*POL*)*P*** and ***D*[*LmpAq6*(*POL*)*C*]*tg***}. Here, A = −CH_2_−, −NH−, O, or S; G = Cl, Br, or I; Z = any group lacking aliphatic carbons.

On the other hand, *DQI*_*a*_(*tg*) descriptors such as ***D*[*LmpAq*1(*V*)*C*]*tg***, ***D*[*LmpAq*6(*POL*)*C*]*tg***, and ***D*[*LmpAq*5(*POL*)*P*]*tg*** describe the importance of controlling the steric factors (Figure 4). Thus, ***D*[*LmpAq*1(*V*)*C*]*tg*** expresses the increase of property *V* of any two atoms (at least one of them being an aliphatic carbon) placed at the topological distance of one. This is the third most significant descriptor and its value can be increased by augmenting the number of aliphatic carbons in the molecule. In case that the number of aliphatic carbons is low, these atoms should be attached to others with relatively high bulkiness (e.g., Cl, Br, and I). In terms of the number of aliphatic carbons that should exist in the molecules, the descriptor ***D*[*LmpAq*6(*POL*)*C*]*tg*** constrains ***D*[*LmpAq*1(*V*)*C*]*tg***. This is because ***D*[*LmpAq*6(*POL*)*C*]*tg*** (ranked fifth in terms of importance) involves the decrease of the *POL* of any two atoms (one of them being an aliphatic carbon) placed at the topological distance equal to six. Consequently, to decrease the value of this descriptor, the number of aliphatic carbons should be kept to a minimum, and/or the atoms placed at the topological distance of six (or lower) with respect to these aliphatic carbons should be preferably low-polarizability atoms such as fluorine, oxygen, and in less degree, nitrogen.

In the case of the descriptor ***D*[*LmpAq*5(*POL*)*P*]*tg*** (the sixth most influential descriptor), this characterizes the augmentation of the *POL* of any two atoms (at least one of them must be an aromatic carbon) which are placed at the topological distance of five. The value of this molecular descriptor can be increased by raising the number of aromatic carbons and/or placing bulky atoms such as halogens (except for fluorine) at topological distances of five or three with respect to the aromatic carbons.

**FIGURE 5:**
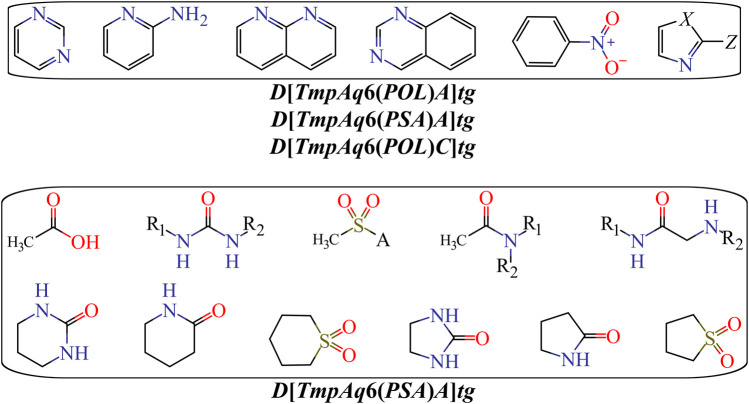
Fragments whose presence positively increase the POL {***D*[*LmpAq6*(*POL*)*A*]*tg***} or the PSA {***D*[*LmpAq6*(*PSA*)*A*]*tg***}. Here, A = −NH_2_, −OH, or R (alkyl group); X = −NH−, O, or S; Z = Cl, Br, I, or −SH; substituents R_1_ and/or R_2_ can be H, alkyl or aryl groups.

Finally, Figure 5 depicts different types of fragments; some of them have a positive influence on ***D*[*LmpAq*6(*POL*)*A*]*tg*** while others favorably augment the value of ***D*[*LmpAq*6(*PSA*)*A*]*tg***. In this sense, the descriptor ***D*[*LmpAq*6(*POL*)*A*]*tg*** is the most important descriptor in the mt-QSAR-MLP model and represents the increase of the *POL* of any two atoms (at least one of them must be a hydrogen bond acceptor) placed at the topological distance equal to six but also lower distances such as two or three. As most of the atoms able to act as hydrogen bond acceptors (N, O, and F) have very low *POL*, then, their neighbor atoms at the aforementioned topological distance should have high polarizabilities (e.g., Cl, Br, I, S, an aromatic carbon, or pyridinic nitrogen). On the other hand, the descriptor ***D*[*LmpAq*6(*PSA*)*A*]*tg*** follows the same line of thinking in terms of the topological distance and the type of atoms involved. Nevertheless, ***D*[*LmpAq*6(*PSA*)*A*]*tg*** focuses on the augmentation of the *PSA*, being the second most influential descriptor.

We would like to point out that although each *DQI*_*a*_(*tg*) descriptor offers information regarding a defined physicochemical property combined with a specific structural aspect, it must not be expected that the infinitely (seemingly desirable) variation in the values of the *DQI*_*a*_(*tg*) descriptors will conduct to an increase in the inhibitory activity. Noticed that, as explained above, some *DQI*_*a*_(*tg*) descriptors are constrained by others. Therefore, only the joint interpretation of the *DQI*_*a*_(*tg*) descriptors in the mt-QSAR-MLP model will provide how, through the introduction of certain molecular fragments, these descriptors can vary harmoniously so a molecule will comply with the structural requirements needed to exhibit multi-target activity against the five proteins reported in this study. The joint interpretation of the descriptors in the mt-QSAR-MLP model indicates that the aromatic and heteroaromatic rings (at least two) can be present in any region. Aliphatic chains and rings (including their heteroatom-based counterparts) can also appear in different parts of a molecule but preferably attached to both aromatic (or heteroaromatic) rings and bulky atoms (e.g., Cl, Br, I, S, and P). Halogens must also be kept in the periphery of the molecules. At least two functional groups containing atoms capable of acting as hydrogen bond acceptors (or donors) must be present, being also close (topological distance lower than 6) to the aforementioned bulky atoms and/or attached to aromatic carbons; if two or more polar functional groups formed by at least two atoms are present, they must be as distant as possible one from the other.

### Virtual Design of Multi-Target Inhibitors Against Parasitic Proteins

Here, we experimented by following a series of guidelines reported recently, which enable the virtual design of new molecules with multi-target activity (Kleandrova et al., 2016; Speck-Planche et al., 2016; Speck-Planche and Cordeiro, 2017a; Speck-Planche and Cordeiro, 2017b; Speck-Planche, 2018; Speck-Planche, 2019; Speck-Planche and Scotti, 2019). The purpose of the experiment was to demonstrate that although the presence of certain fragments is important for the appearance and/or enhancement of the multi-target activity, how these fragments are connected between each other will principally define whether a molecular can simultaneously inhibit different parasite proteins.

**FIGURE 6:**
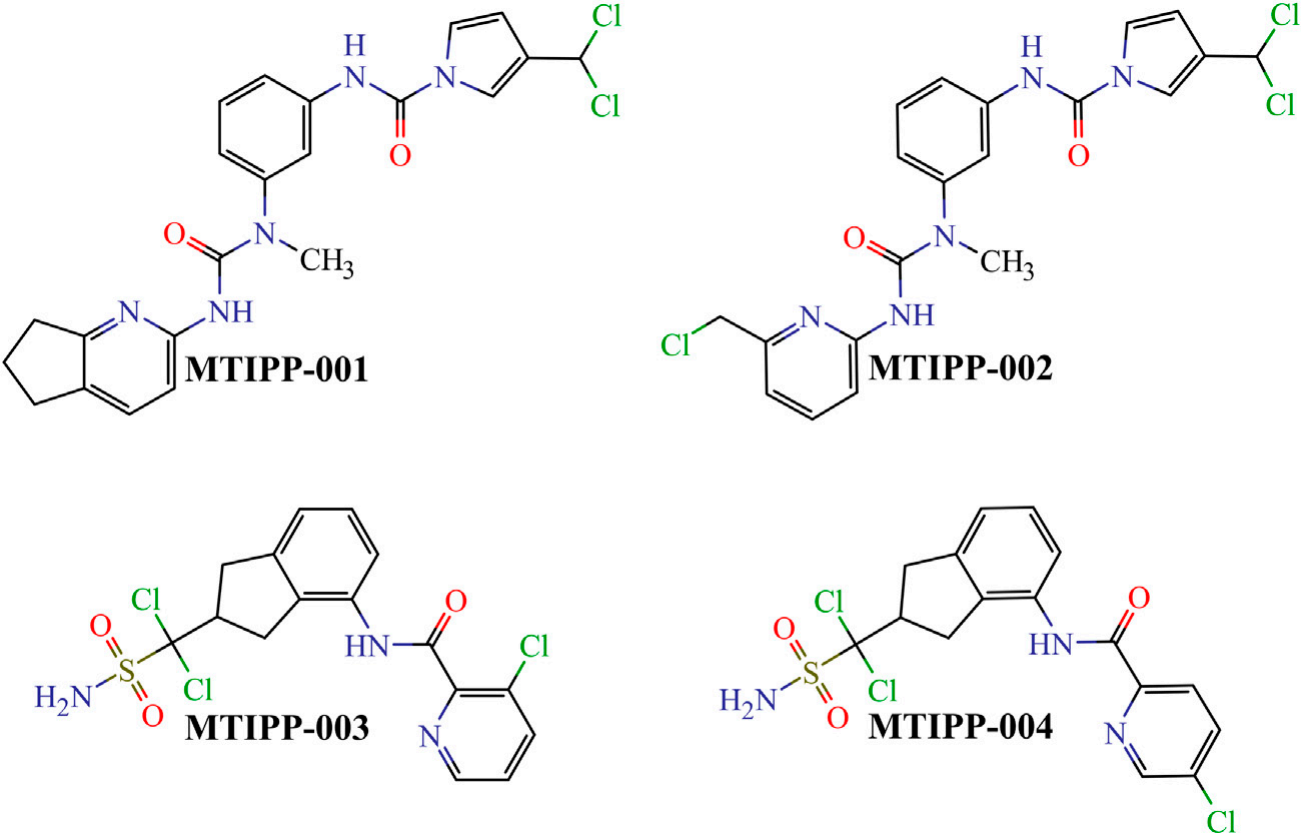
Chemical structures of the molecules designed and predicted by using the mt-QSAR-MLP model.

By rigorously following the joint interpretation of the *DQI*_*a*_(*tg*) descriptors in the mt-QSAR-MLP model, we designed four molecules belonging to two different chemical families (Figure 6). While doing so, we assembled the molecules by connecting or fusing different molecular fragments considered to positively contribute to the desirable variations in the values of the *DQI*_*a*_(*tg*) descriptors.

We would like to emphasize that when referring to the potential inhibitory activity of any of the designed molecules against a given parasitic protein, we do it always by considering the corresponding cutoff (IC_50_) value of inhibitory activity reported in this work. Thus, according to the results of the predictions performed by the mt-QSAR-MLP mode (Table 4), all the designed molecules were predicted as multi-target inhibitors of at least three of the five parasite proteins reported in this study. All the predictions fell within the applicability domain of the mt-QSAR-MLP except for those belonging to the inhibitory activity of the molecules MTIPP-001 and MTIPP-002 against the protein glycylpeptide N-tetradecanoyltransferase (*T. brucei brucei*). More details regarding the calculated *DQI*_*a*_(*tg*) descriptors, the predictions of the designed molecules, and the assessment of the applicability for these can be found in Supplementary Material S4.

**TABLE 4:** Molecules designed and the predictions of their multi-target profiles against the parasite proteins.

ID	*Pred*_*IA*_*i*_(*tg*)^a^ ^,^ ^b^				
	Plasmepsin 2 (*P. falciparum*)	DHODH (*P. falciparum*)	Cruzipain (*T. cruzi*)	DHFR (*T. gondii*)	GPNTDT (*T. brucei brucei*)
MTIPP-001	1	1	−1	1	−1
MTIPP-002	1	1	1	1	−1
MTIPP-003	1	1	1	1	1
MTIPP-004	1	1	1	1	1

^**a**^ ***Pred_IA***_***i***_ **(*tg*)**—Predicted value of the categorical variable of inhibitory activity [***IA***_***i***_ **(*tg*)**].

^**b**^ The abbreviations DHODH, DHFR, and GPNTDT stand for the proteins named dihydroorotate dehydrogenase, dihydrofolate reductase, and glycylpeptide N-tetradecanoyltransferase, respectively.

If we inspect MTIPP-001 and MTIPP-002, it will be easy to see the remarkable similarity between their chemical structures. The only difference is that the cyclopentane moiety fused with the pyridinic ring in MTIPP-001 is replaced by the chloromethyl moiety in MTIPP-002. Yet, this small change is responsible for the differences in the multi-target profiles of these two molecules. Although the aforementioned replacement leads to a detrimental decrease of the value of the descriptor ***D*[*LmpAq*1(*V*)*C*]*tg*** (which benefits from the increment of aliphatic carbons), it desirably increases the values of ***D*[*LmpAq*6(*POL*)*A*]*tg*** and ***D*[*LmpAq*5(*POL*)*P*]*tg***, also favorably decreasing ***D*[*LmpAq*6(*POL*)*C*]*tg***. These three *DQI*_*a*_(*tg*) descriptors account for the fact while MTIPP-001 has been predicted to inhibit three proteins, its analog MTIPP-002 may be able to inhibit four of these biomolecular targets.

In contrast to MTIPP-001 and MTIPP-002, the molecules MTIPP-003 and MTIPP-004 present sulfonamide moiety which has a considerably higher *PSA* than any of the other functional groups. Furthermore, in MTIPP-003 and MTIPP-004, both fragments sulfonamide and amide are closer to the chlorines. This arrangement of atoms, which also includes the correct positioning of the aliphatic portions with respect to both aromatic carbons and chlorines particularly causes the dramatic (favorable) increment of the values of the descriptors ***D*[*LmpAq*6(*POL*)*A*]*tg***, ***D*[*LmpAq*6(*PSA*)*A*]*tg***, and ***D*[*LmpAq*1(*V*)*C*]*tg***; these are the top three *DQI*_*a*_(*tg*) descriptors, exhibiting the highest influence/discriminatory power in the mt-QSAR-MLP model. Consequently, these *DQI*_*a*_(*tg*) descriptors are the main responsible for the fact that MTIPP-003 and MTIPP-004 were predicted as multi-target inhibitors against the five parasite proteins reported in this work.

Considering their potential multi-target activity, the designed molecules were searched in different databases such as ChEMBL and ZINC (Irwin and Shoichet, 2005). The aim here was to check if these molecules are reported in the scientific literature. When searching for similar compounds, the similarity cutoff was ≥0.7. Under this condition, all the designed molecules seem to be new, as no results of similar molecules were found.

### Docking Calculations Suggest the Multi-Target Potential of the Designed Molecules

As depicted in Table 5, each protein was docked against four organic compounds. The first of them is the reference ligand, which forms the crystallized complex with the protein. The second organic chemical is present in both the ChEMBL database and our dataset used to build the mt-QSAR-MLP model; the experimental IC_50_ value of that organic chemical is equal to the activity cutoff selected for each protein. Notice that these ChEMBL organic chemicals offer a point of comparison to estimate the inhibitory activity of any query molecule by considering the different cutoffs of activity associated with the parasitic proteins. We also docked the designed molecules MTIPP-002 and MTIPP-004, which belong to different chemical families. In the case of MTIPP-002, we selected it over its analog MTIPP-001 because the former was predicted by the mt-QSAR-MLP model to inhibit 4 out of 5 parasitic proteins; MTIPP-001 was predicted as active against only three proteins. We also chose MTIPP-004 over its analog MTIPP-003 because MTIPP-004 was predicted slightly better against two of the parasitic proteins according to their posterior probabilities; in one protein, MTIPP-003 was predicted better than MTIPP-004 and for the other two remaining proteins, MTIPP-003 and MTIPP-004 had the same value of predicted probabilities (see Supplementary Material S4).

**TABLE 5:** Results from the docking calculations.

PDB ID^a^	Protein (Organism)^b^	Ligand ID^c^	Energy (kcal/mol)	RMSD^d^
2BJU	Plasmepsin 2 (*P. falciparum*)	IH4	−172.78	0.25
		CHEMBL3264802	−153.7	-
		MTIPP-002	−150.21	-
		MTIPP-004	−103.99	-
6I55	DHODH (*P. falciparum*)	DZB	−110.4	0.67
		CHEMBL1784557	−80.8	
		MTIPP-002	−120.5	
		MTIPP-004	−70.9	
1ME3	Cruzipain (*T. cruzi*)	P10	−151.93	0.33
		CHEMBL565866	−95.77	-
		MTIPP-002	−145.7	-
		MTIPP-004	−96.48	-
4KY4	DHFR (*T. gondii*)	1UE	−123.8	0.15
		CHEMBL145528	−103.4	-
		MTIPP-002	−150.9	-
		MTIPP-004	−114.2	-
HM_6QD9	GPNTDT (*T. brucei brucei*)	CHEMBL3959734	−67.8	-
		MTIPP-002	−111.7	-
		MTIPP-004	−110.22	-

^a^ The ID written as “HM_6QD9” indicates that the 3D structure of the protein was obtained via homology modeling by using the protein’s amino acid sequence from the PDB ID 6QD9 as the template.

^b^ The abbreviations DHODH, DHFR, and GPNTDT stand for the proteins named dihydroorotate dehydrogenase, dihydrofolate reductase, and glycylpeptide N-tetradecanoyltransferase, respectively.

^c^ For each protein (PDB ID), the ligands are ordered in the following manner: **1)** the reference ligand, **2)** compound from the dataset used to build the mt-QSAR-MLP model and whose IC_50_ value is equal to the cutoff of activity selected for each protein, **3)** ligand belonging to the first chemical family of designed molecules, and **4)** ligand belonging to the second chemical family of designed molecules. ^d^RMSD is the root-mean-square deviation of the atomic coordinate during the redocking of the reference ligands.

In general, the preliminary results of the docking calculations depicted in Table 5 converge with the results of the predictions performed by the mt-QSAR-MLP model regarding the multi-target profile of the designed molecules. We, however, observed divergencies in the proteins plasmepsin 2 (MTIPP-002 and MTIPP-004 suggested as inactive) and dihydroorotate dehydrogenase (MTIPP-004 indicated as inactive). This comes from the fact that the molecular docking calculations and QSAR modeling (e.g., the mt-QSAR-MLP model developed here) ‘catch’ different physicochemical and structural information regarding the chemical diversity and complexity of the molecules when inhibiting proteins. Therefore, these two computational techniques can be used in a complementary manner to study the biological profiles of the molecules in the context of protein inhibition.

Another detail that can be extracted from Table 5 is that for the molecule MTIPP-002, the mt-QSAR-MLP and the docking calculations converge in 4 out of 5 parasitic proteins in the sense that this designed molecule is a multi-target inhibitor. Notice that the binding energy for MTIPP-002 is lower than those of the ChEMBL organic chemicals. Similar behavior occurs for the case of MTIPP-004 in 3 out of 5 parasitic proteins. Interestingly, regardless of the protein, the docking calculations suggest that, according to the energy values, MTIPP-002 is more active than MTIPP-004 although the latter was predicted by the mt-QSAR-MLP model to inhibit the five parasitic proteins while MTIPP-002 was predicted as active against only four proteins. However, there is no contradiction because while the docking calculations can be used to compare if one molecule is more active than the other, the mt-QSAR-MLP model only predicts if a molecule will be active or inactive against a protein by considering a defined cutoff value.

At the structural level, the results from the docking calculations are provided in Supplementary Material S5, which illustrates the different protein-ligands interactions. Thus, here, for the case of each parasitic protein, we will compare the designed molecules MTIPP-002 and MTIPP-004 with ChEMBL chemicals in terms of the strength and number of interactions that help explain the results obtained in Table 5. In doing so, we will focus only on the parasitic proteins where the docking calculations converge (either partially or totally) with the predictions performed by the mt-QSAR-MLP model. These proteins are dihydroorotate dehydrogenase (*P. falciparum*), as well as cruzipain (*T. cruzi*), dihydrofolate reductase (*T. gondii*), and glycylpeptide N-tetradecanoyltransferase (*T. brucei brucei*). Our objective is to demonstrate that the designed molecules MTIPP-002 and MTIPP-004 are more active than the corresponding chemicals represented in Table 5.

We would like to highlight that in some cases, some unfavorable interactions were observed (marked in red color in the upcoming figures). We do not discard the possibility that these interactions may be associated with the computational algorithm employed to perform the docking calculations but we prefer a plausible phenomenological explanation. This is related to the fact that none of the ChEMBL chemicals present sufficiently optimized structures to effectively interact with the different amino acids in the binding site of each parasitic proteins. Nevertheless, these ChEMBL chemicals have experimental IC_50_ values in the submicromolar range against their corresponding parasitic proteins. On the other hand, the molecules MTIPP-002 and MTIPP-004 were designed as potential multi-target inhibitors. This means that because of the very different physicochemical and structural characteristics of the binding sites of the parasitic proteins, it is very probable that they will not cause strong inhibition as in the case of a specific/mono-target inhibitor. However, as MTIPP-002 and MTIPP-004 were designed to inhibit most of the parasitic proteins at the submicromolar range, this could translate into a much higher inhibition of the growth of the parasitic species when compared with a mono-target inhibitor. Following, we will discuss the interactions that mainly contribute to the stability/instability of the different protein-ligand complexes.

**FIGURE 7:**
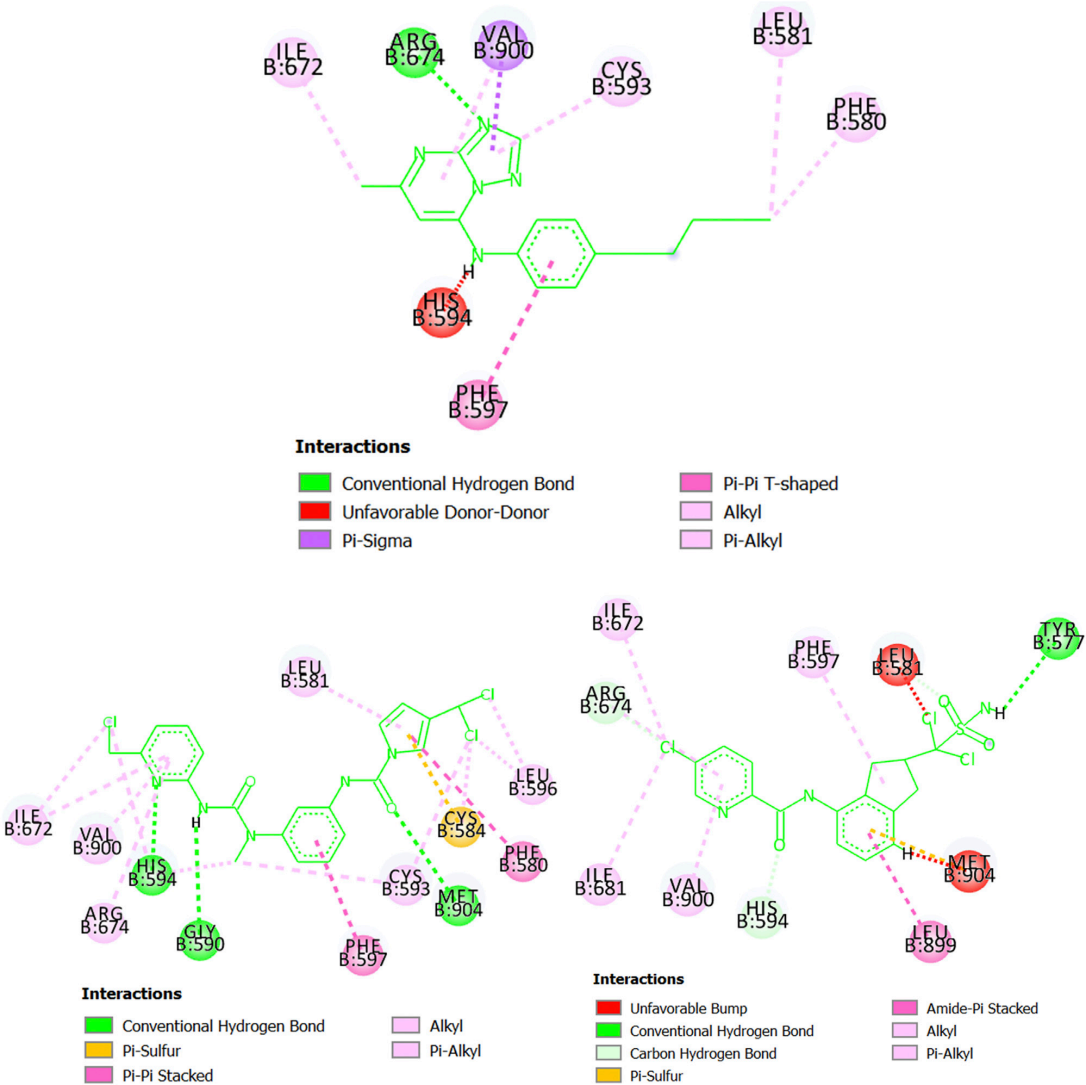
Diagram depicting the interactions of CHEMBL1784557 **(top center)**, MTIPP‐002 **(left bottom)** and MTIPP‐004 **(right bottom)** with dihydroorotate dehydrogenase (*P. falciparum*).

Starting with the protein dihydroorotate dehydrogenase (*P. falciparum*), it can be seen that one of the pyridinic nitrogen atoms in the five-membered ring of the molecule CHEMBL1784557 (experimental IC_50_ = 820 nM) interacts with the residue Arg674 via hydrogen bond (Figure 7). Also, the ring itself makes contact with Val900 and Cys593 through pi-sigma and pi-alkyl interactions, respectively. The benzene ring of CHEMBL1784557 also contributes to stabilizing the complex by interacting with Phe507 (pi-pi T-shaped). Other interactions involve the two methyl groups of CHEMBL1784557. Yet, due to proximity, a repulsion-based interaction takes place between the secondary amine of CHEMBL1784557 and the residue His594, which is detrimental to the stability of the protein-ligand complex. In contrast, the molecule MTIPP-002 forms a hydrogen bond with His594, as well as with Gly590 and Met904. These hydrogen bonds together with pi-pi stacked (Phe580 and Phe597) and pi-sulfur (Cys584) are the main contributors to the stability of the complex formed by MTIPP-002 and dihydroorotate dehydrogenase (*P. falciparum*), which has lower energy than that formed by the same protein and CHEMBL1784557. This suggests that the inhibitory potency of MTIPP-002 is greater than that of CHEMBL1784557. In the case of the molecule MTIPP-004, it forms a hydrogen bond with Tyr577, as well as other interactions such as pi-sulfur (Met904), amide-pi stacked (Leu899). Despite these and other several interactions such as alkyl, pi-alkyl, carbon-hydrogen bonds, MTIPP-004 presents two bumps due to steric hindrance with Leu581 and Met904, which decreases the stability of the complex, thus diminishing the ability of MTIPP-004 to strongly inhibit dihydroorotate dehydrogenase (*P. falciparum*) at the submicromolar concentration of 820 nM (activity cutoff based on IC_50_).

**FIGURE 8:**
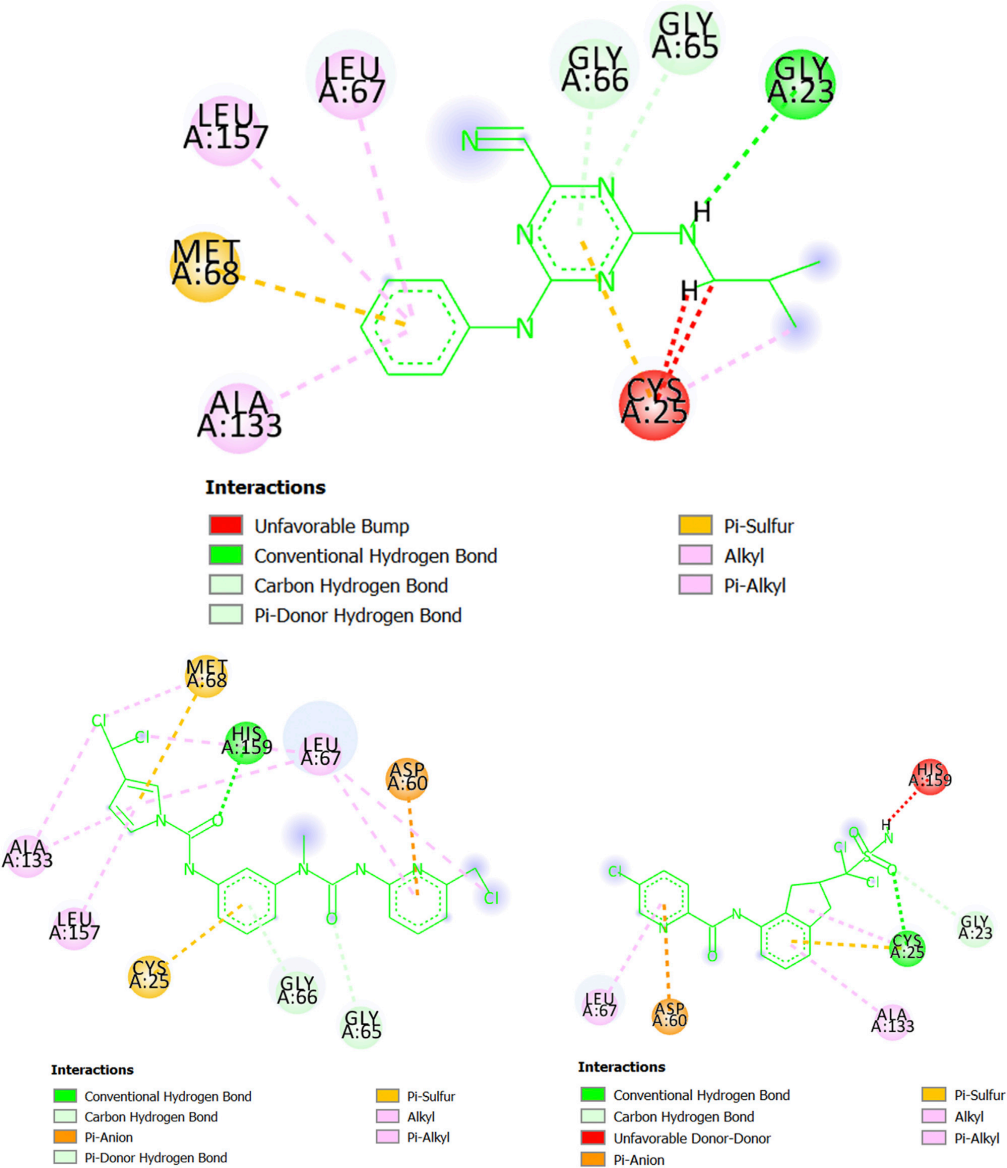
Interactions of CHEMBL565866 **(top center)**, MTIPP‐002 **(left bottom)** and MTIPP‐004 **(right bottom)** with cruzipain (*T. cruzi*).

Regarding cruzipain (*T. cruzi*), we can observe in Figure 8 that the molecule CHEMBL565866 (experimental IC_50_ = 890 nM) forms a hydrogen bond with the residue Gly23 while also having pi-sulfur interactions with Met68 and Cys25. Simultaneously, CHEMBL565866 is involved in other interactions such as pi-alkyl, pi-donor hydrogen bond, and carbon-hydrogen bond. This molecule has a double repulsive interaction with Cys25, which considerably decreases the stability of the complex formed between CHEMBL565866 and cruzipain (*T. cruzi*). In this context, the molecule MTIPP-002 has several interactions, including those with the residues His159 (hydrogen bond), Asp60 (pi-anion), and Cys25 and Met68 (both pi-sulfur) which are the main energetic contributors to the complex stability. These interactions, together with those involving the three chlorine atoms, the pyrrolic ring, and other moieties, indicate that MTIPP-002 should have a higher inhibitory potency (lower IC_50_ value) than CHEMBL565866. In the case of MTIPP-004, despite having an unfavorable (donor-donor) contact with His159, it greatly compensates by interacting with the residues Cys25 (hydrogen bond, pi-sulfur, and alkyl-alkyl), Asp60 (pi-anion), Leu67, and Ala133 (pi-alkyl), as well as Gly23 (carbon-hydrogen bond). For the case of the complex cruzipain-MTIPP-004, these interactions lead to an energy value lower than that estimate for the complex cruzipain-CHEMBL565866. This suggests that MTIPP-004 should have IC_50_ ≤ 890 nM.

**FIGURE 9:**
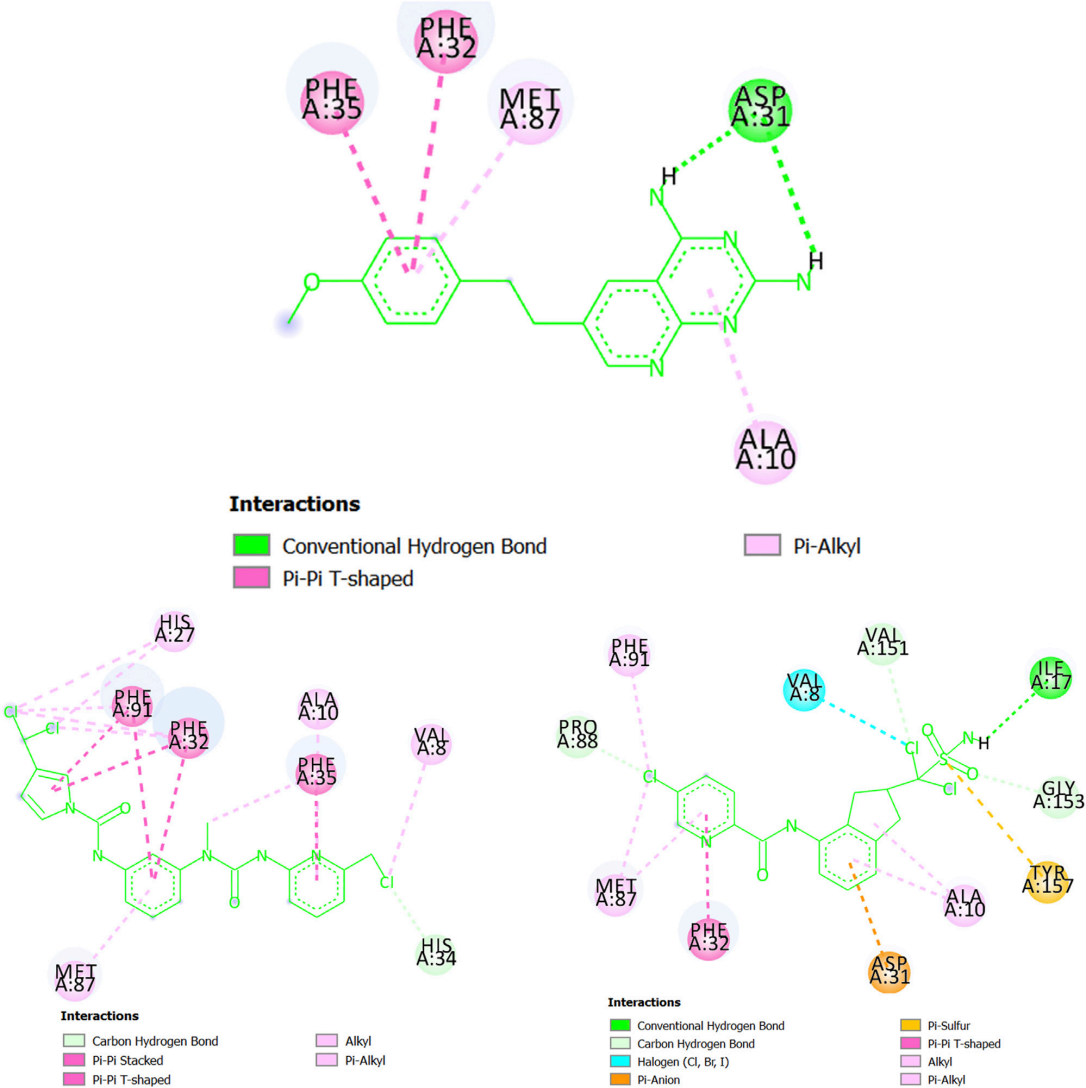
Chemicals interacting with dihydrofolate reductase (*T. gondii*): CHEMBL145528 **(top center)**, MTIPP-002 **(left bottom)** and MTIPP-004 **(right bottom)**.

Following with the protein dihydrofolate reductase (*T. gondii*), in Figure 9 we have several regions of CHEMBL145528 (experimental IC_50_ = 250 nM) interacting via a double hydrogen bond (Asp31), pi-alkyl associations (Ala10 and Met87), and pi-pi T-shaped configurations (Phe32 and Phe35). Notice, however, that the two hydrogen bonds with Asp31 lack directionality (see the 3D view in Supplementary Material S5), and therefore they may be relatively weak. Interestingly, MTIPP-002, despite lacking hydrogen bond, forms a huge number of hydrophobic interactions such as pi-pi stacked and pi-pi T-shaped configurations with the amino acids Phe32, Phe35, and Phe91. In this sense, it has been experimentally demonstrated that the presence of simultaneous pi-interactions of a molecule with the residues Phe32 and Phe91 are essential in achieving inhibitory potency (IC_50_) at the submicromolar range (Welsch et al., 2016). On the other hand, there is also a great number of alkyl-alkyl and pi-alkyl interactions where the amino acids Val8, Ala10, His27, and Met87 participate; His34 is involved in a carbon-hydrogen bond. A key aspect of the interactions of MTIPP-002 with the different amino acids is that most of them seem highly directional, which, for the complex formed by this molecule and dihydrofolate reductase (*T. gondii*), yield an energy value lower than the complexes formed by the same protein with either CHEMBL145528 or MTIPP-004. Therefore, MTIPP-002 should have an IC_50_ ≤ 250 nM. For the case of MTIPP-004, this molecule should also be expected to exhibit IC_50_ ≤ 250 nM since the stability of the complex MTIPP-004- dihydrofolate reductase (*T. gondii*) is favored over that of the complex CHEMBL145528-dihydrofolate reductase (*T. gondii*). This is due to the presence of adequate interactions of MTIPP-004 with the amino acids Ile17 (hydrogen bond), Val8 (halogen bond), Asp31 (pi-anion), Phe32 (pi-pi T-shaped), and Tyr157 (pi-sulfur). There are also other favorable interactions involving alkyl groups (either from MTIPP-004 or the amino acids) and carbon-hydrogen bonds.

**FIGURE 10:**
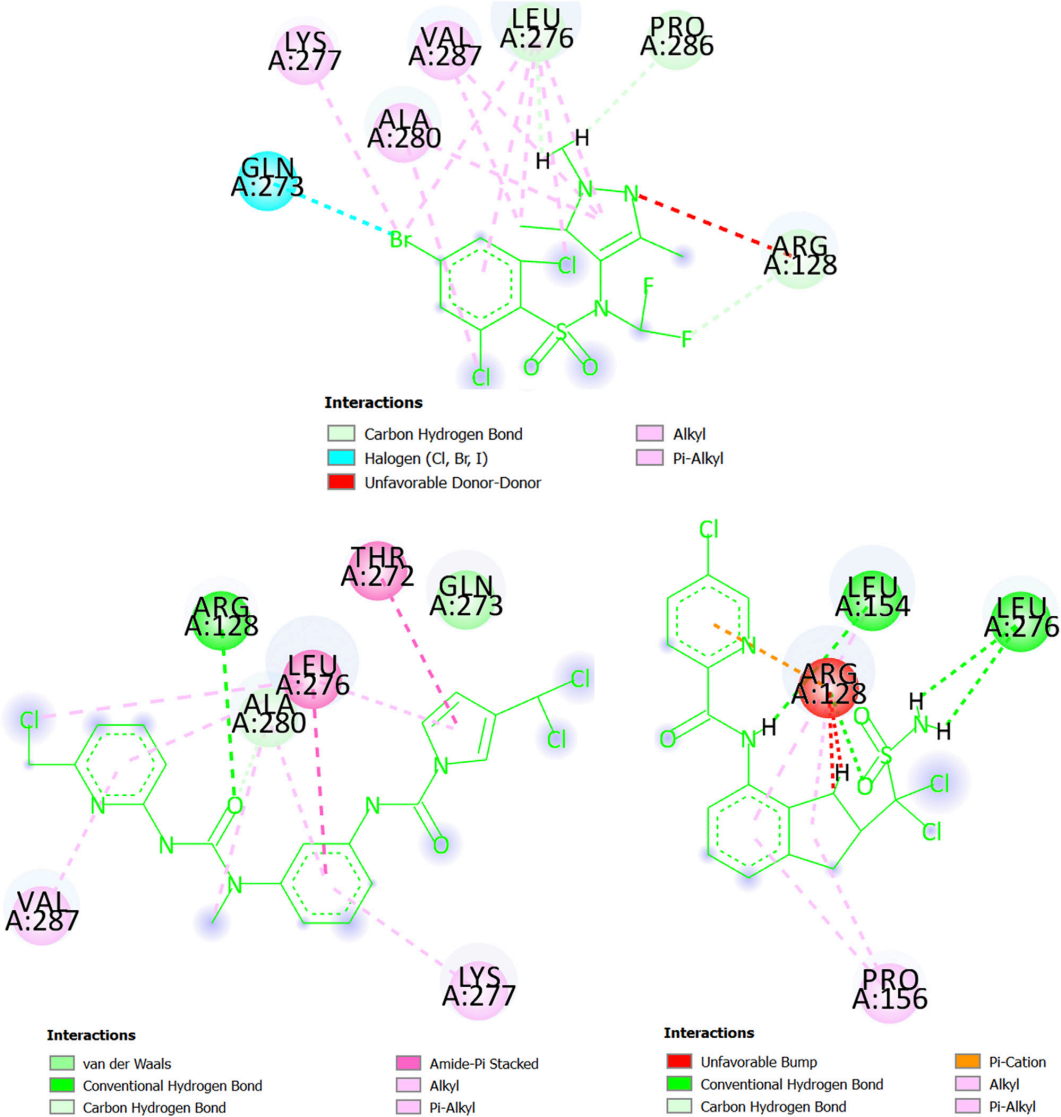
Amino acids of the binding site of glycylpeptide N-tetradecanoyltransferase (*T. brucei brucei*) interacting with CHEMBL3959734 **(top center)**, MTIPP-002 **(left bottom)** and MTIPP-004 **(right bottom)**.

Last, we have the glycylpeptide N-tetradecanoyltransferase (*T. brucei brucei*) whose 3D structure was created by using homology modeling. The steps and results of the homology modeling for this protein can be found in Supplementary Material S6. The 3D structure of glycylpeptide N-tetradecanoyltransferase (*T. brucei brucei*) is now freely available at https://swissmodel.expasy.org/repository/uniprot/Q388H8 as part of the SWISS-MODEL Repository (Bienert et al., 2017), which is a database of annotated 3D protein structure models. In Figure 10, the pi-alkyl interactions (and others involving alkyl groups or halogens) between the chemical CHEMBL3959734 (experimental IC_50_ = 270 nM) and glycylpeptide N-tetradecanoyltransferase prevail, particularly with the residues Leu276, Ala280, and Val287 (Lys277 participates in less degree). There are also three carbon-hydrogen bonds (Arg128, Leu276, and Pro286) and a halogen bond with the residue Gln273. In any case, Arg128 is present in an unfavorable donor-donor interaction with CHEMBL3959734. At the same time, we can deduct from Figure 10 that both MTIPP-002 and MTIPP-004, form more stable complexes with glycylpeptide N-tetradecanoyltransferase (*T. brucei brucei*) than CHEMBL3959734. From one side, MTIPP-002 forms a hydrogen bond with Arg128, the same amino acid the unfavorable influences the stability of the complex formed by CHEMBL3959734 and glycylpeptide N-tetradecanoyltransferase (*T. brucei brucei*). Besides, MTIPP-002 exhibits relatively strong amide-pi stacked interactions with the residues Thr272 and Leu276; the latter also participates in pi-alkyl (together with Ala280 and Val287) and other interactions based on the presence alkyl group (together with Ala280). Other non-covalent interactions can also be observed. All these interactions point out to the direction of considering MTIPP-002 to have IC_50_ ≤ 270 nM. On the other hand, in the case of MTIPP-004, there is detrimental energetic contribution because of the repulsion with Arg128 although the same residue favorably present in a hydrogen bond, and pi-cation, pi-alkyl, and alkyl-alkyl interactions. Anyway, MTIPP-004 counterbalances by forming other three hydrogen bonds, one with Leu154 and two with Leu276; Leu154 also participates in a pi-alkyl interaction together with Pro156 (also involved in an alkyl-alkyl interaction). All these interactions help to explain why the energy value obtained for MTIPP-002 and MTIPP-004 are very similar when interacting with s for the complexes of glycylpeptide N-tetradecanoyltransferase (*T. brucei brucei*). Consequently, MTIPP-004 is also expected to have IC_50_ ≤ 270 nM.

## Druglikeness and Synthetic Accessibility

We examined the four designed molecules in terms of their compliance with Lipinski’s rule of five (Lipinski et al., 2001), the Ghose’s filter (Ghose et al., 1999), and the Veber’s rule (Veber et al., 2002). These guidelines are based on the estimation of a series of physicochemical properties that permit to analyze of the druglikeness of any molecule, in particular, their capacity to exhibit a good oral bioavailability. The physicochemical properties were calculated by the program AlvaDesc v1.0.14 (Alvascience-Srl, 2019) and included the number of hydrogen bond donors (*HBD*), the number of hydrogen bond acceptors (*HBA*), the molecular weight (*MW*), the logarithm of the partition coefficient octanol/water (*logP*), the number of atoms (*nAT*), the molar refractivity (*MR*), the number of rotatable bonds (*RBN*), and the *PSA*. A report of these properties for the designed molecules can be found in Supplementary Material S7; the physicochemical properties of the molecules designed here are in agreement with Lipinski’s rule of five and the other variants. We also employed the webserver SwissADME to estimate the synthetic accessibility of the designed molecules. In this sense, SwissADME predicts the synthetic accessibility score (*SAS*), which ranges from 1 (easily synthesizable) to 10 (difficult to synthesize). The *SAS* values for the designed molecules ranges from 3.23 to 3.48 (Supplementary Material S7). Considering the closeness to 1 of these *SAS* values, it can be deduced that the designed molecules should be relatively easy to synthesize.

## Concluding Remarks

A more efficient eradication of many parasitic diseases can in principle be achieved with the use of multi-target inhibitors. The fast search of such a class of antimicrobial therapeutics depends in great part on the power and accuracy of modern computational tools. The mt-QSAR-MLP built in this work model represents an advance in early drug discovery against parasitic diseases because with this *in silico* tool and the theoretical support provided by the molecular docking calculations, it is possible to rationally design potential antiparasitic agents by simultaneously inhibiting diverse targets involved in the virulence and/or survival of several pathogenic parasites. The present report confirms the promising applications of the mt-QSAR approaches, which can be extended to many therapeutic areas.

## Data Availability

The original contributions presented in the study are included in the article/SupplementaryMaterial, further inquiries can be directed to the corresponding author/s.
