# Supplementary material for: QSAR Modeling for Multi-Target Drug Discovery: Designing Simultaneous Inhibitors of Proteins in Diverse Pathogenic Parasites
Source: Front Chem. 2021 Mar 10;9:634663. doi: 10.3389/fchem.2021.634663 (PMC7987820; doi:10.3389/fchem.2021.634663)
Supplement: Supplementary file 1 [file datasheet1.pdf]

QSAR Modeling for Multi-Target Drug Discovery: Designing Simultaneous Inhibitors of Proteins in Diverse Pathogenic Parasites

Valeria V. Kleandrova[1], Luciana Scotti[2], Francisco Jaime Bezerra Mendonça Junior[3], Eugene Muratov[4], Marcus T. Scotti[2]*, and Alejandro Speck-Planche[2]*

[1]Laboratory of Fundamental and Applied Research of Quality and Technology of Food Production, Moscow State University of Food Production, Volokolamskoe shosse 11, 125080, Moscow, Russian Federation
[2]Postgraduate Program in Natural and Synthetic Bioactive Products, Federal University of Paraíba, 58051-900, João Pessoa, Brazil
[3]Laboratory of Synthesis and Drug Delivery, State University of Paraíba, João Pessoa-PB 58071-160, Brazil
[4]Laboratory for Molecular Modeling, the UNC Eshelman School of Pharmacy, University of North Carolina at Chapel Hill, Chapel Hill, NC 27599, USA

***Correspondence:** Marcus T. Scotti (mtscotti@gmail.com)
Alejandro Speck-Planche (alejspivanovich@gmail.com)

The following content describes the 3D views (left) and 2D diagrams (right) with the interactions between each ligand and its corresponding target.

Protein plasmepsin 2 (*P. falciparum*): PDB ID 2BJU

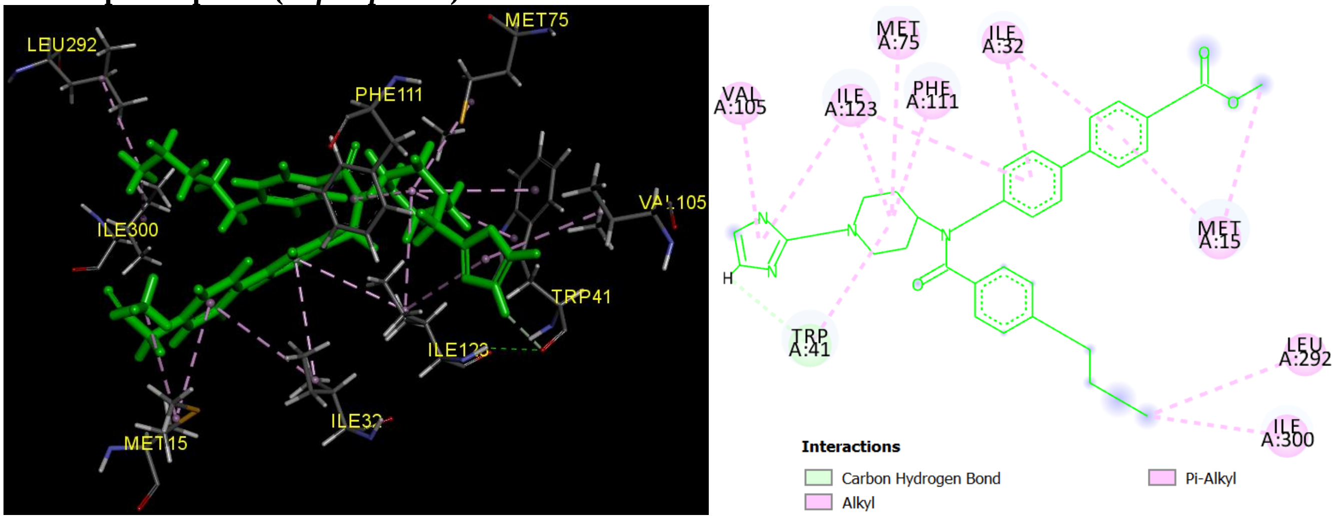

Fig.1S5. Interactions between the IH4 (reference ligand) and the amino acids in the binding site of plasmepsin 2.

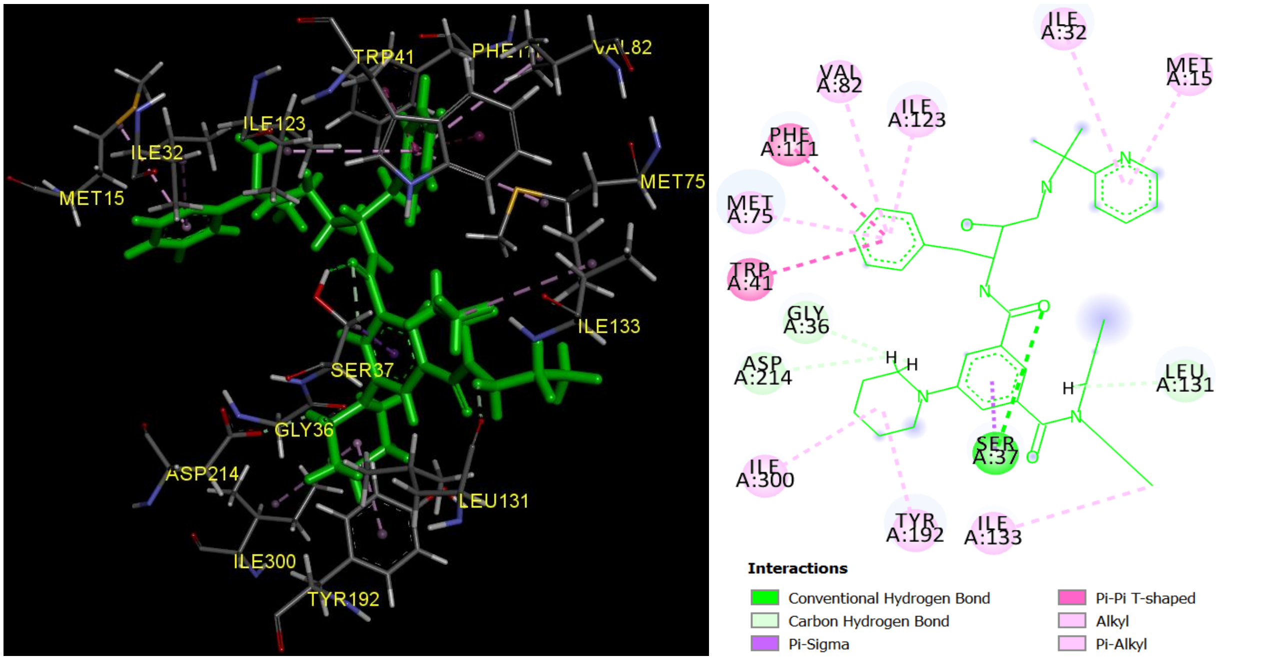

Fig.2S5. Interactions between the molecule CHEMBL3264802 and the amino acids in the binding site of plasmepsin 2.

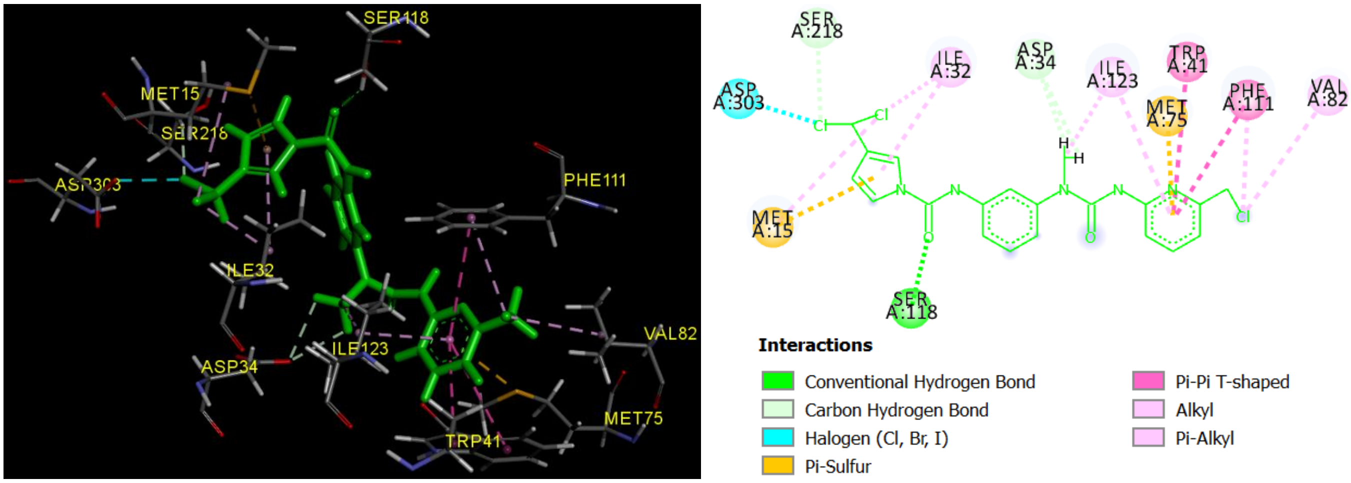

Fig.3S5. Interactions between the molecule MTIPP-002 and the amino acids in the binding site of plasmepsin 2.

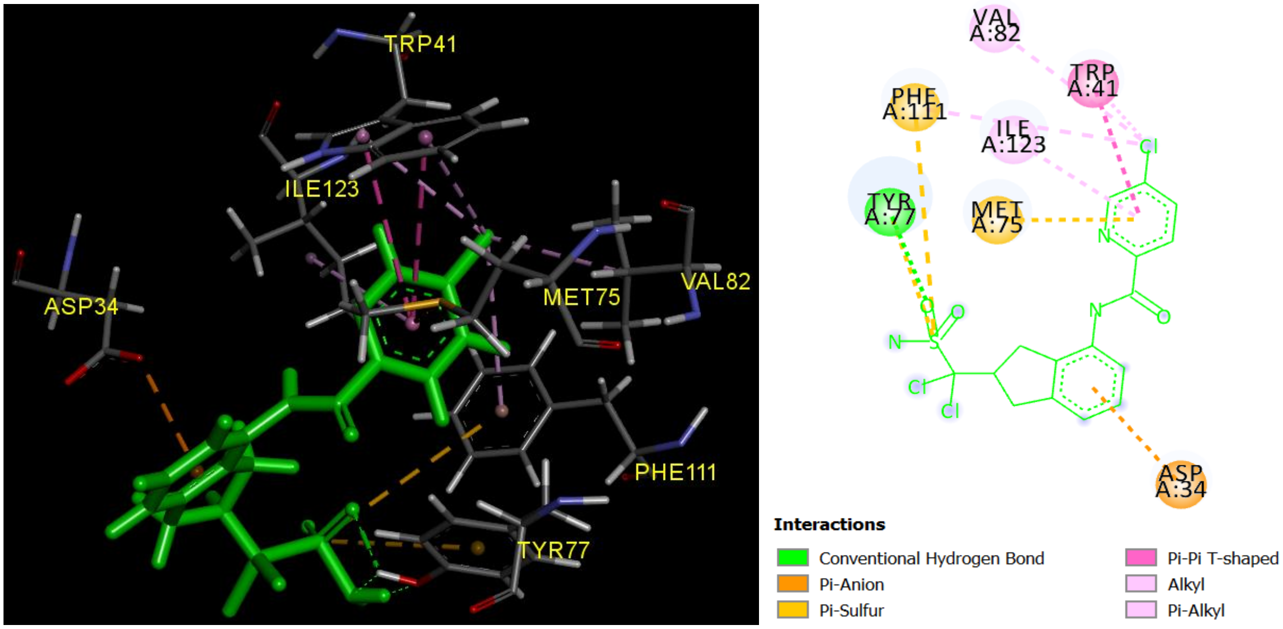

Fig.4S5. Interactions between the molecule MTIPP-004 and the amino acids in the binding site of plasmepsin 2.

Protein dihydroorotate dehydrogenase (*P. falciparum*): PDB ID 6I55

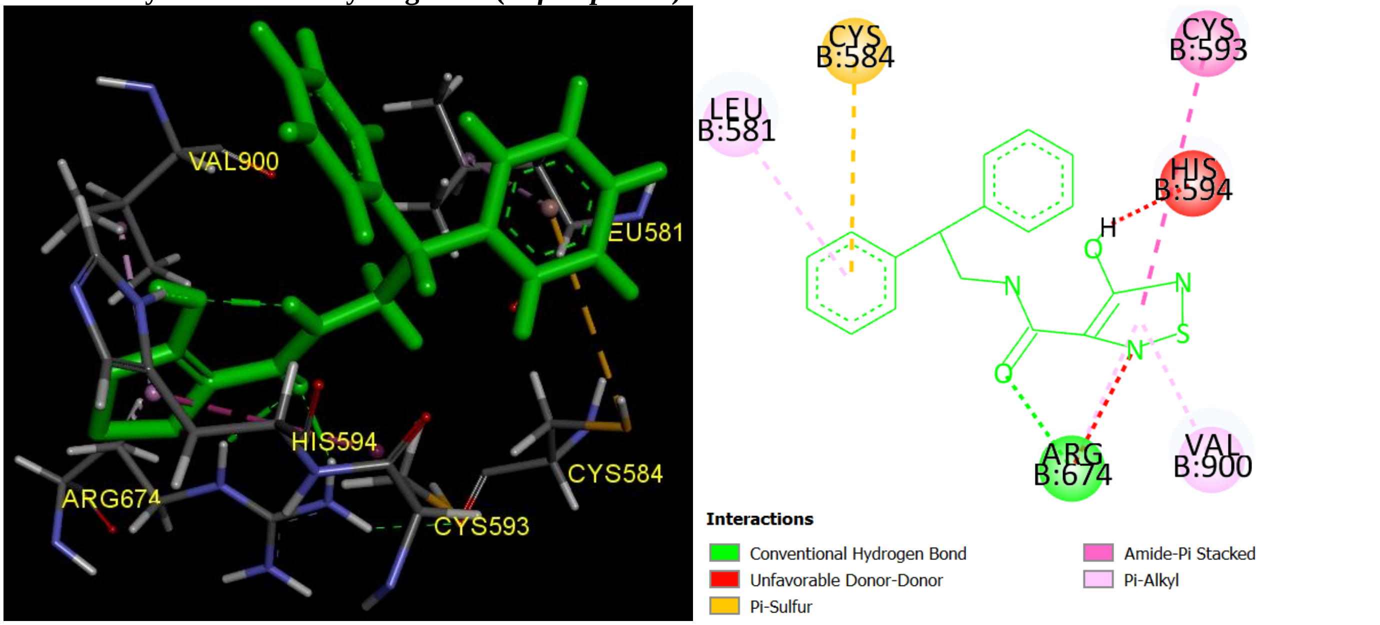

Fig.5S5. Interactions between the DZB (reference ligand) and the amino acids in the binding site of dihydroorotate dehydrogenase.

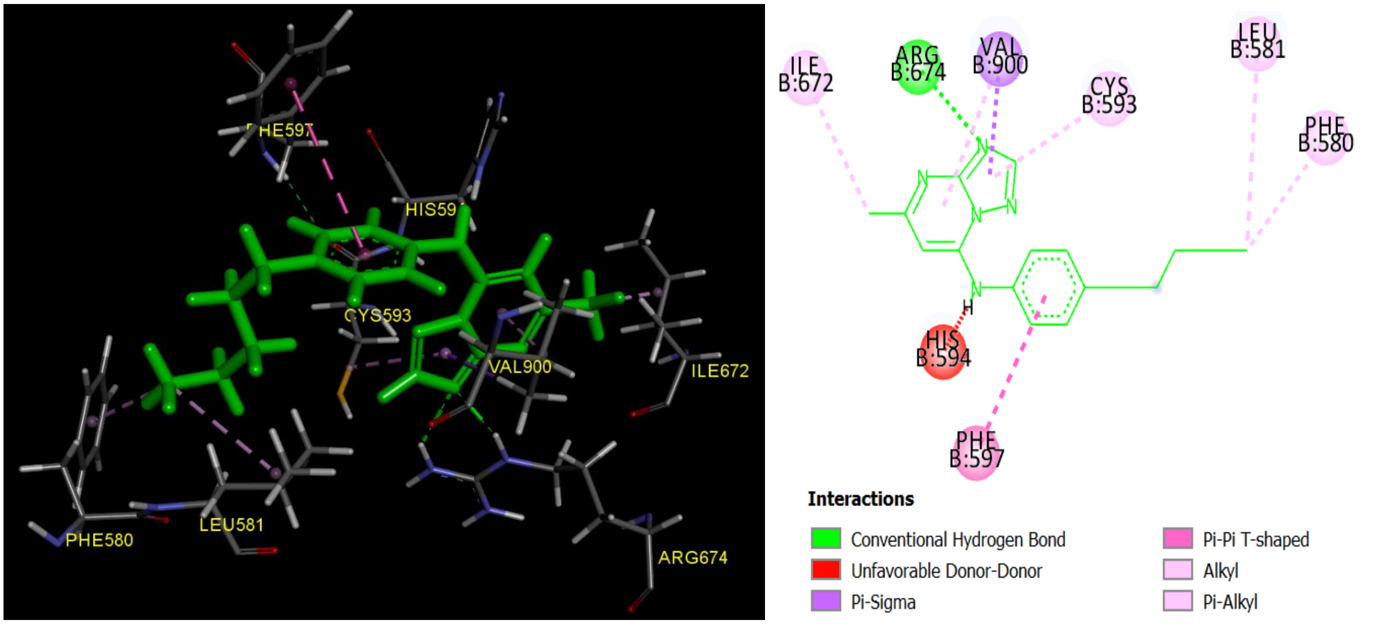

Fig.6S5. Interactions between the molecule CHEMBL1784557 and the amino acids in the binding site of dihydroorotate dehydrogenase.

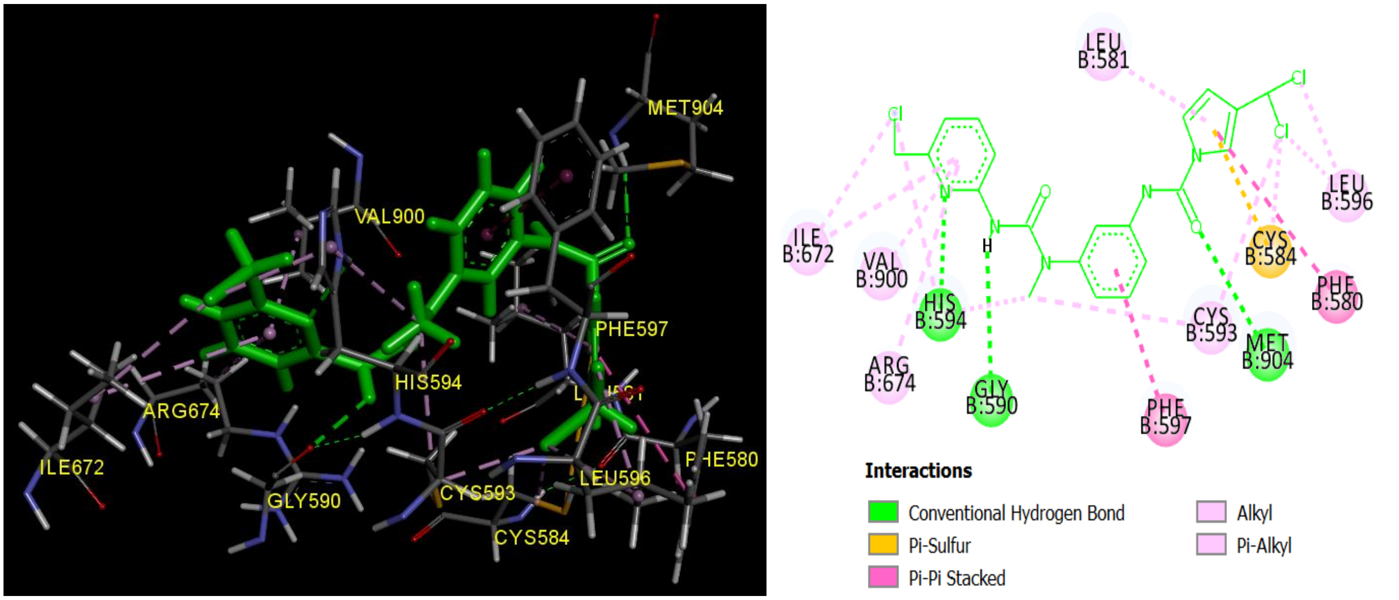

Fig.7S5. Interactions between the molecule MTIPP-002 and the amino acids in the binding site of dihydroorotate dehydrogenase.

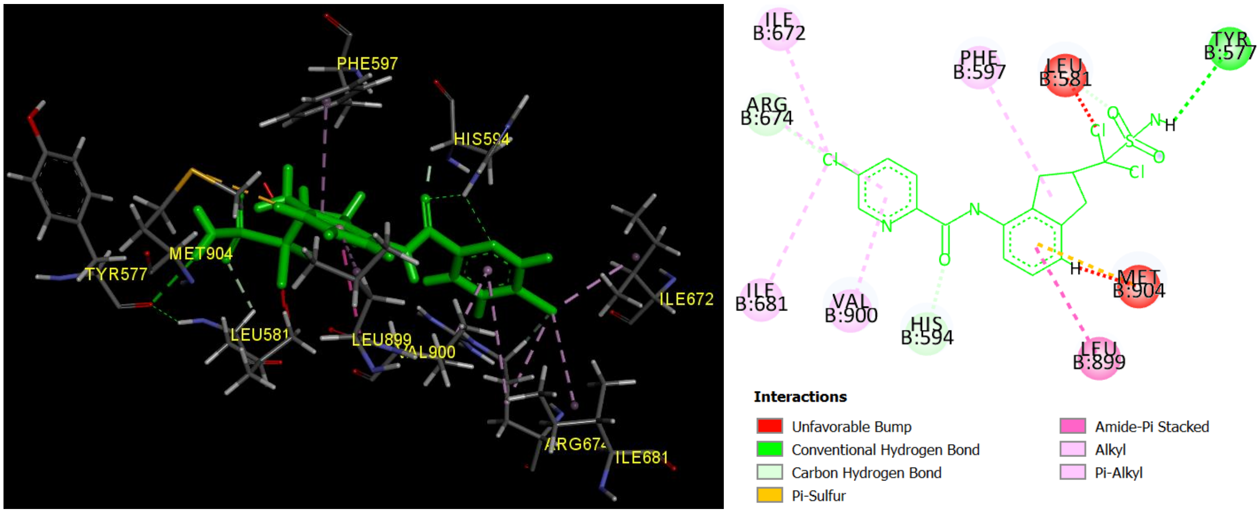

Fig.8S5. Interactions between the molecule MTIPP-004 and the amino acids in the binding site of dihydroorotate dehydrogenase.

Protein cruzipain (*T. cruzi*): PDB ID 1ME3

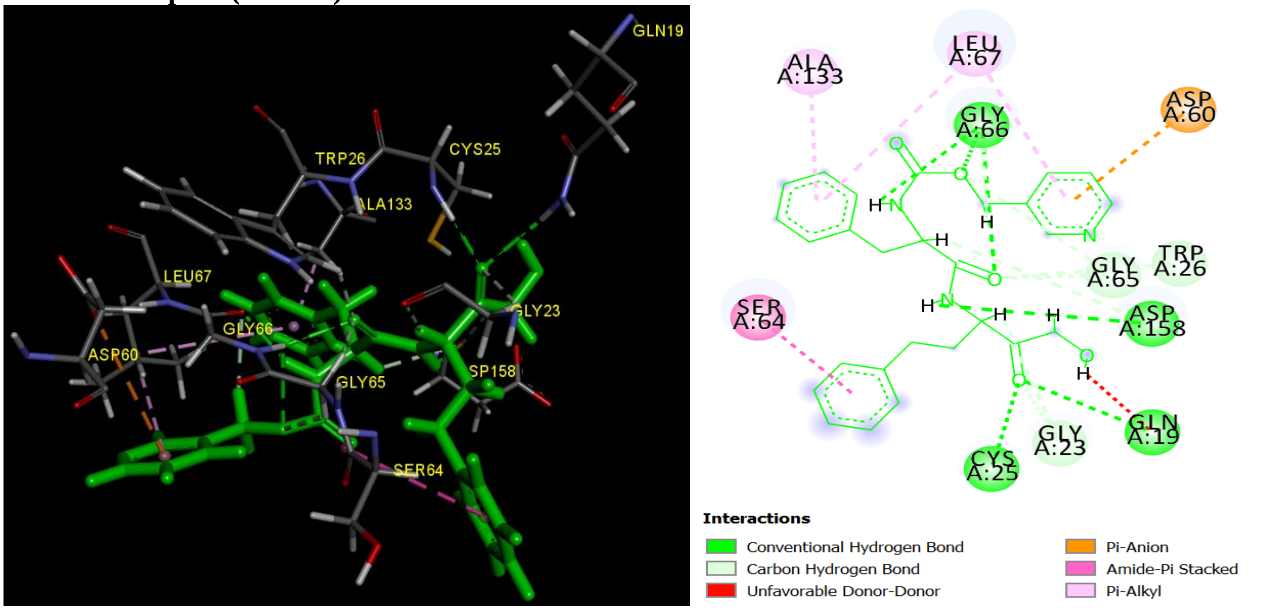

Fig.9S5. Interactions between the molecule P10 (reference ligand) and the amino acids in the binding site of cruzipain.

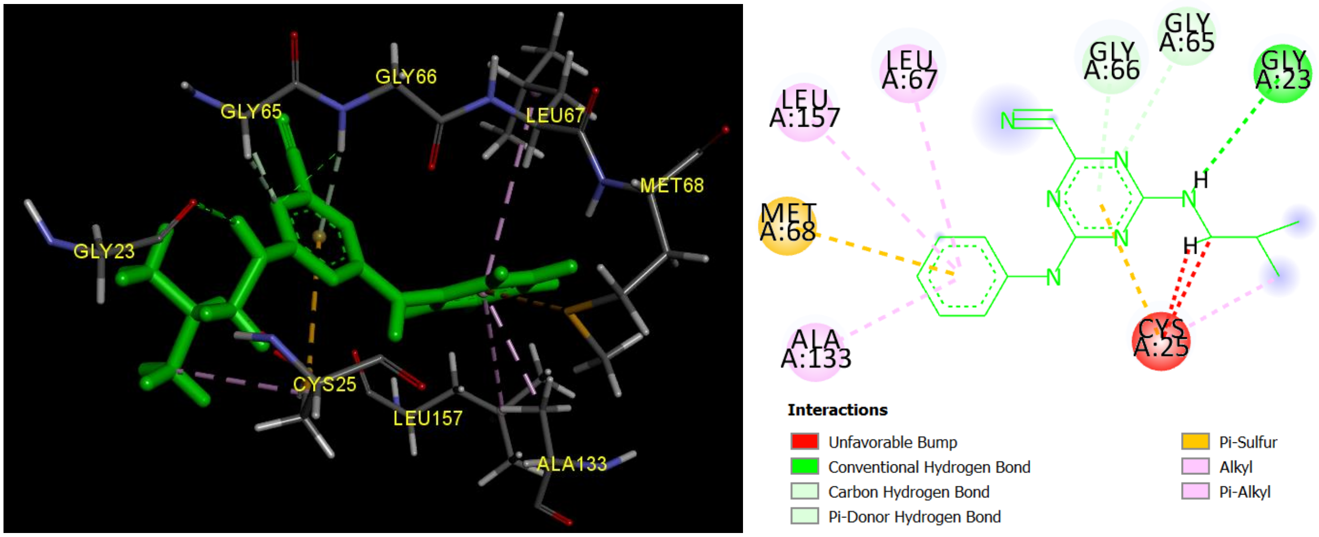

Fig.10S5. Interactions between the molecule CHEMBL565866 and the amino acids in the binding site of cruzipain.

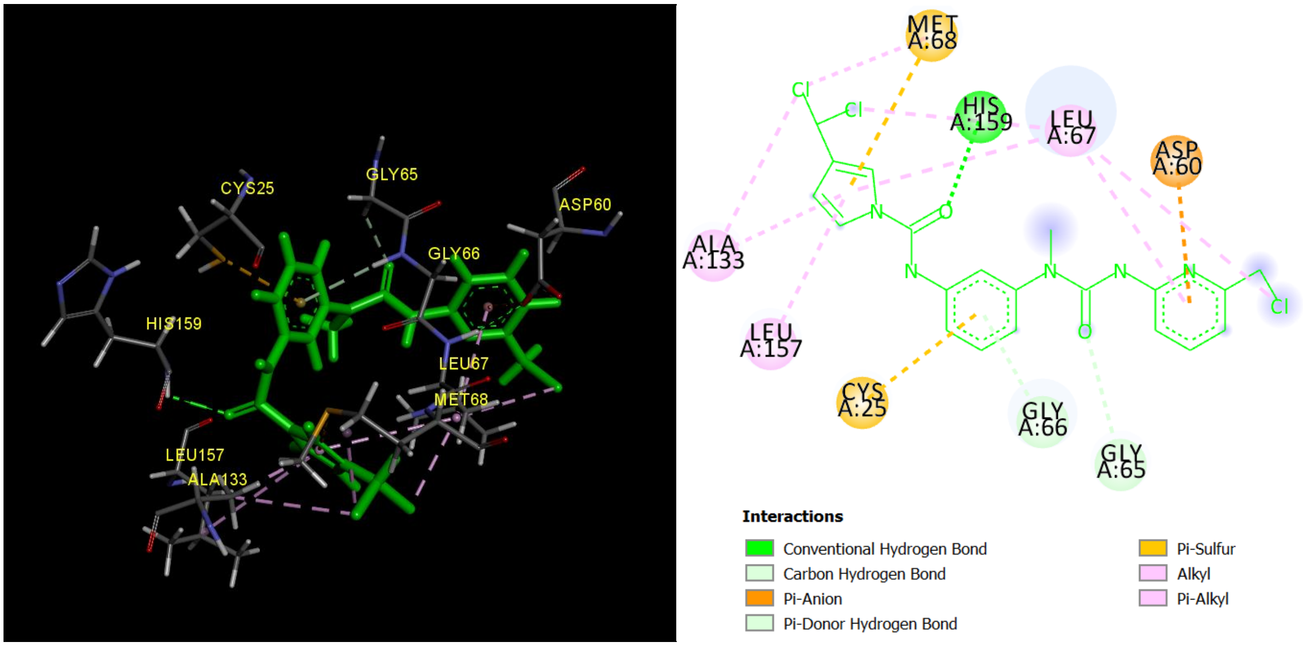

Fig.11S5. Interactions between the molecule MTIPP-002 and the amino acids in the binding site of cruzipain.

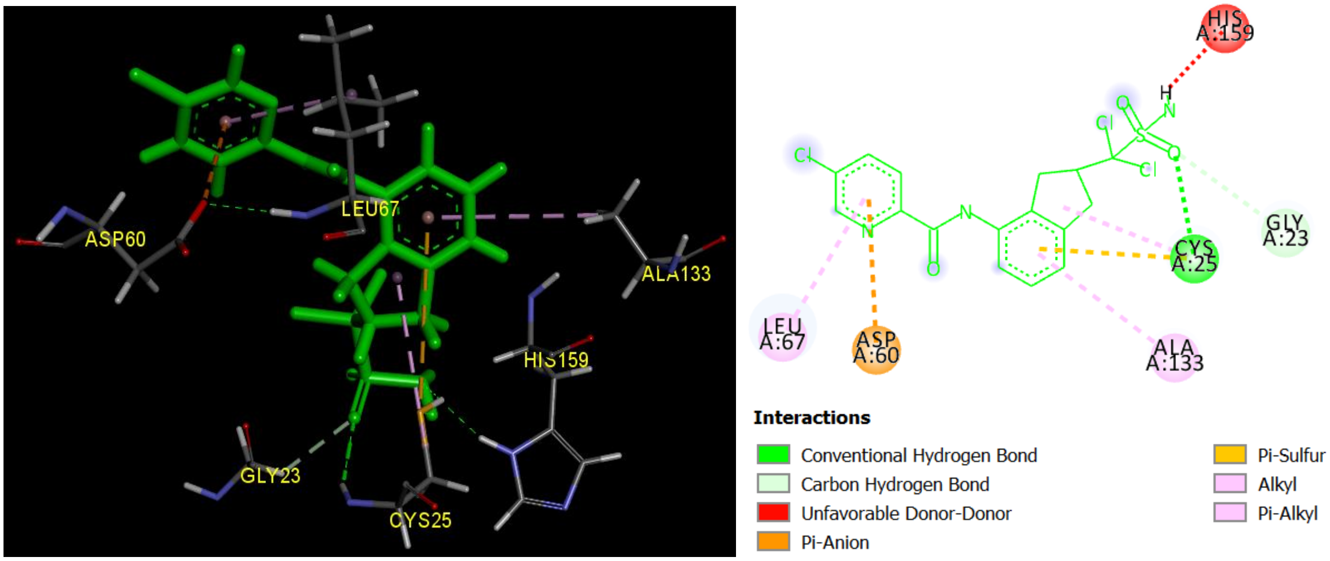

Fig.12S5. Interactions between the molecule MTIPP-004 and the amino acids in the binding site of cruzipain.

Protein dihydrofolate reductase (*T. gondii*): PDB ID 4KY4

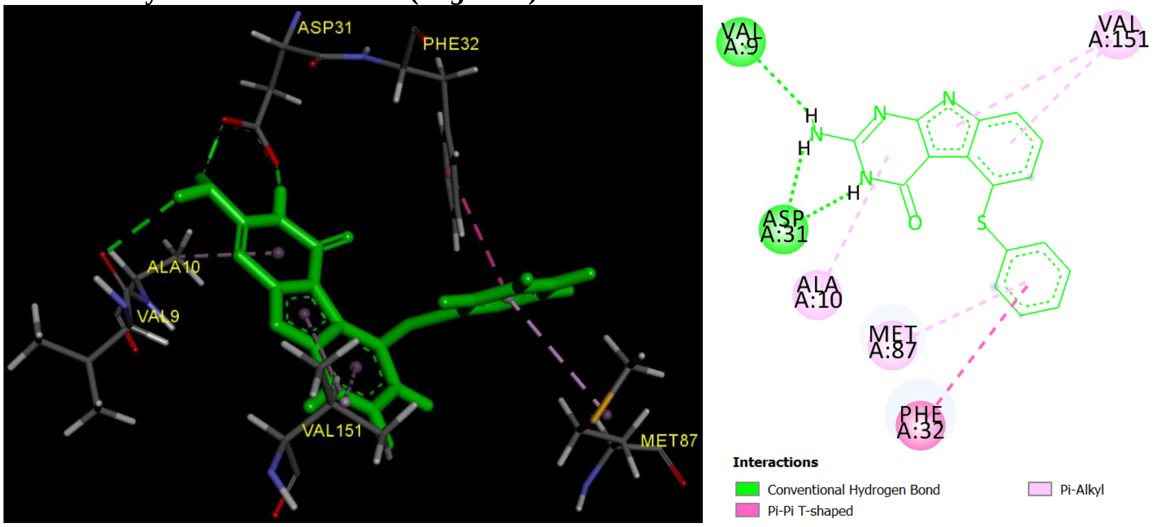

Fig.13S5. Interactions between the 1UE (reference ligand) and the amino acids in the binding site of dihydrofolate reductase.

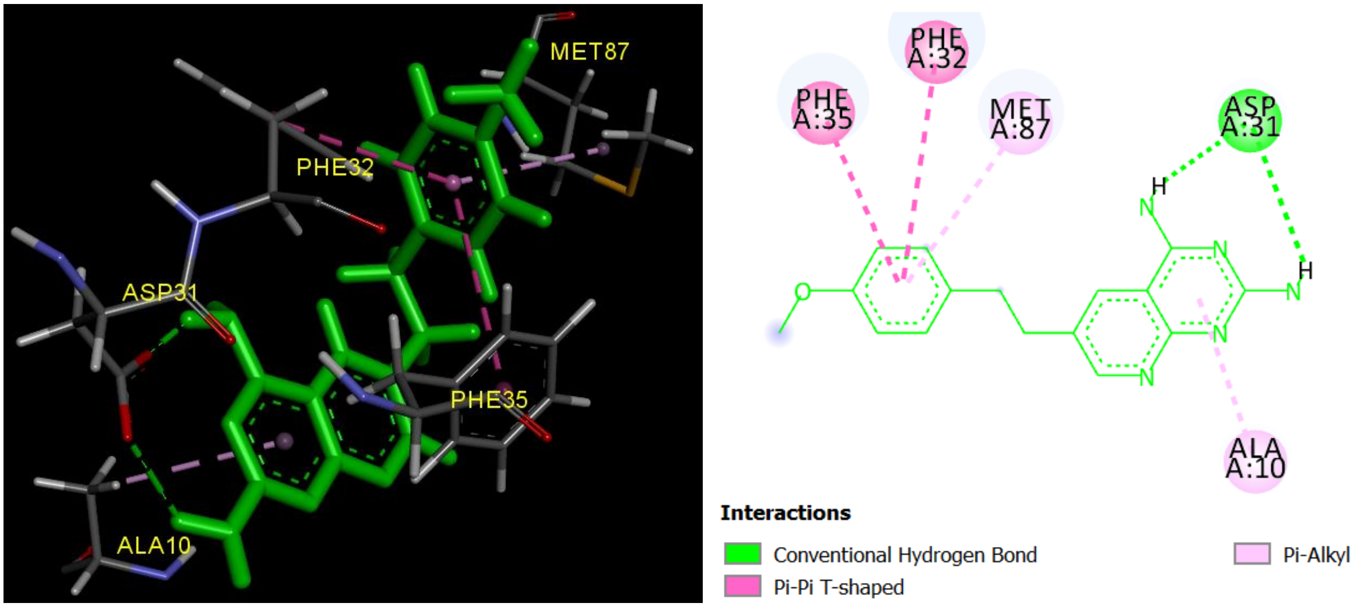

Fig.14S5. Interactions between the molecule CHEMBL145528 and the amino acids in the binding site of dihydrofolate reductase.

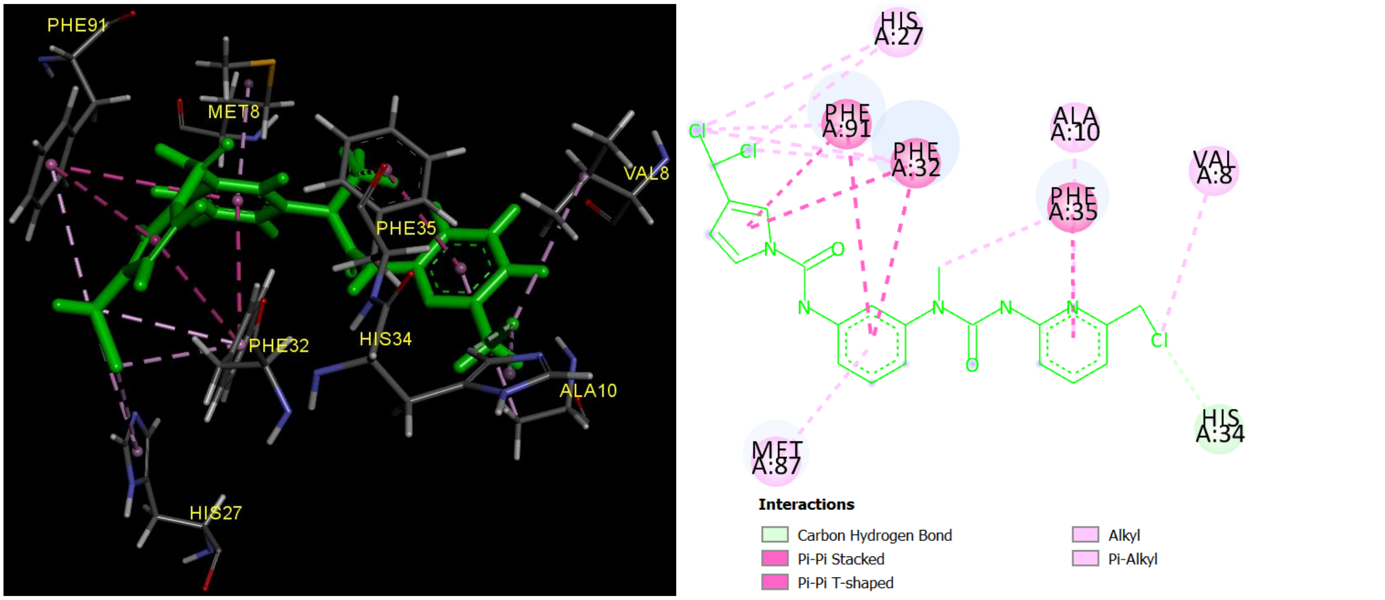

Fig.15S5. Interactions between the molecule MTIPP-002 and the amino acids in the binding site of dihydrofolate reductase.

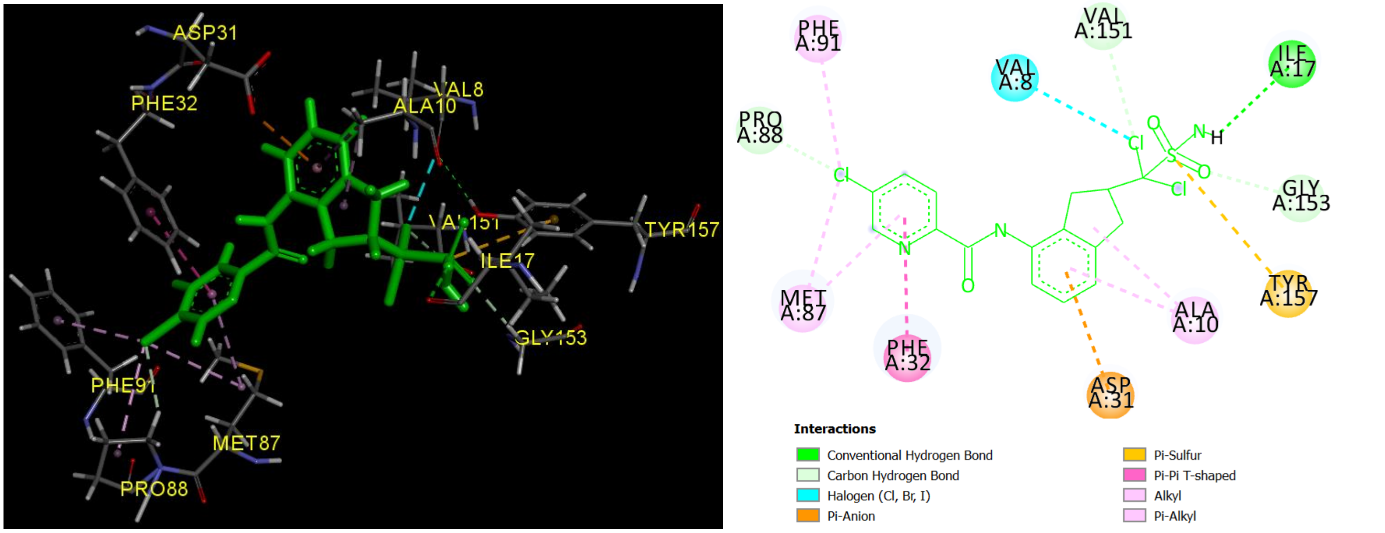

Fig.16S5. Interactions between the molecule MTIPP-004 and the amino acids in the binding site of dihydrofolate reductase.

Protein glycylpeptide N-tetradecanoyltransferase (*T. brucei brucei*)

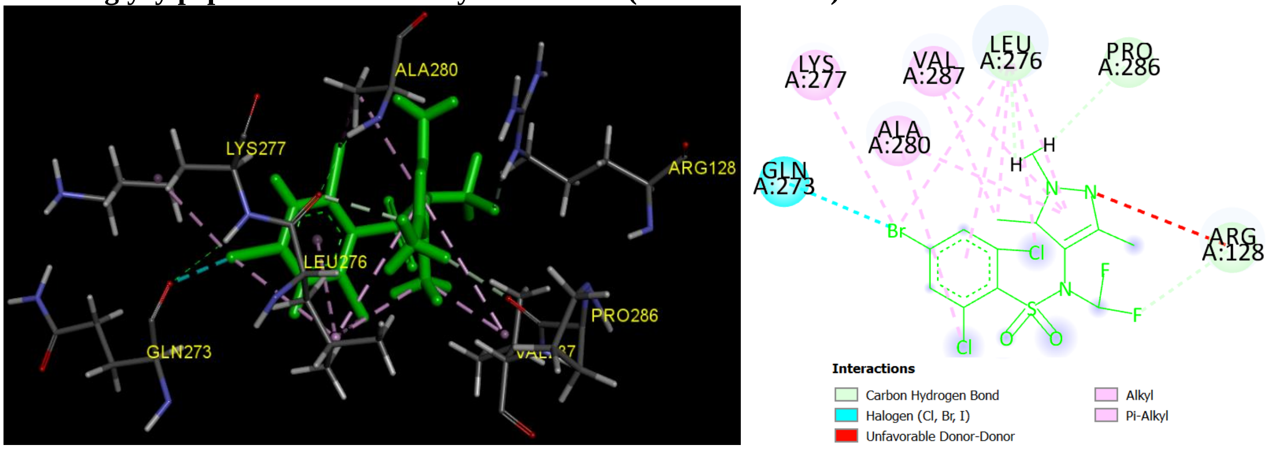

Fig.17S5. Interactions between the molecule CHEMBL3959734 and the amino acids in the binding site of glycylpeptide N-tetradecanoyltransferase.

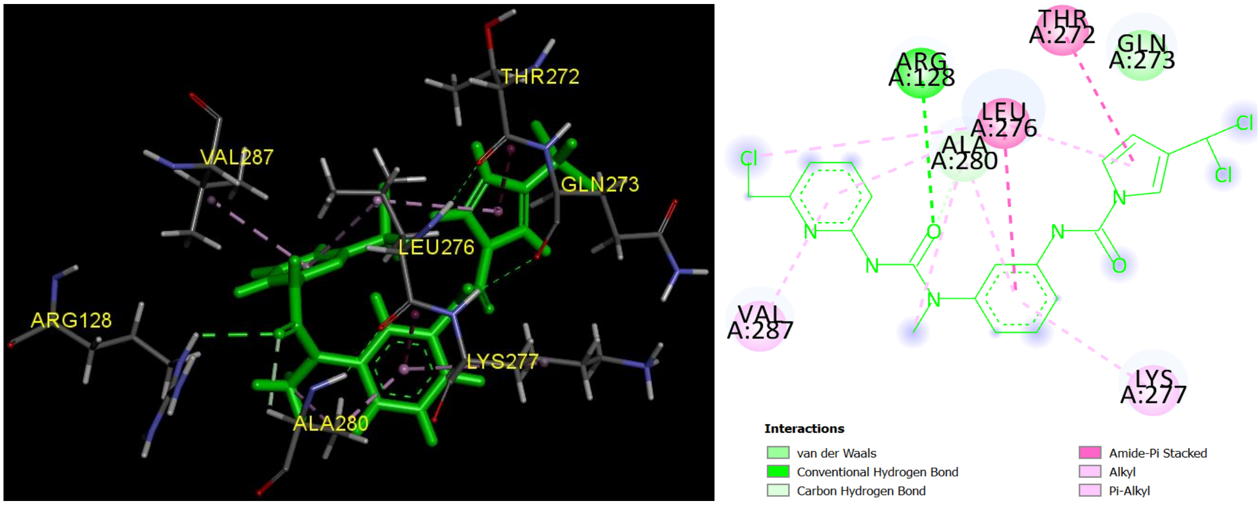

Fig.18S5. Interactions between the molecule MTIPP-002 and the amino acids in the binding site of glycylpeptide N-tetradecanoyltransferase.

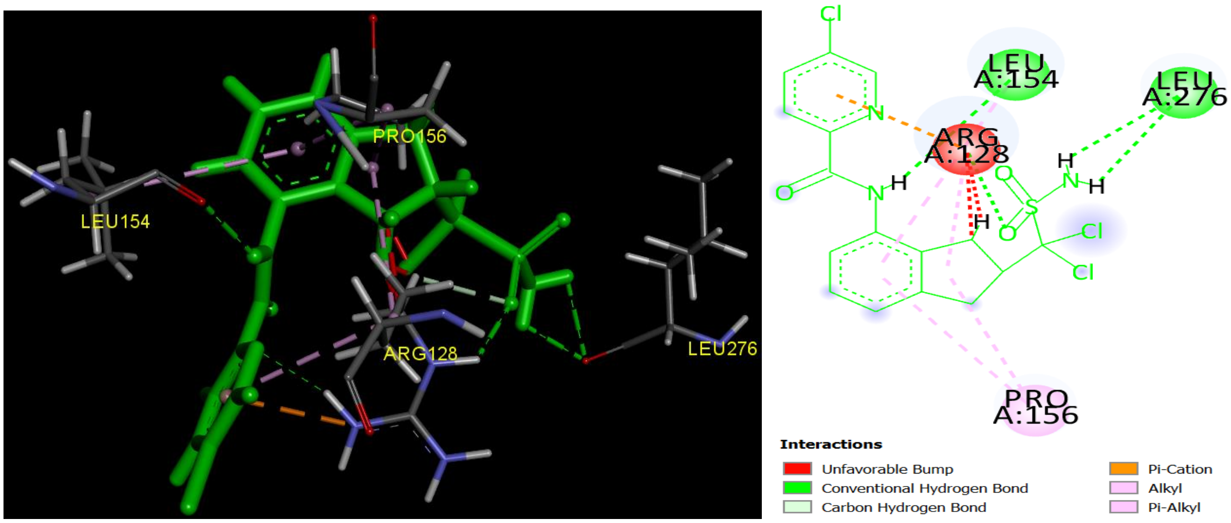

Fig.19S5. Interactions between the molecule MTIPP-004 and the amino acids in the binding site of glycylpeptide N-tetradecanoyltransferase.
