# Supplementary material for: QSAR Modeling for Multi-Target Drug Discovery: Designing Simultaneous Inhibitors of Proteins in Diverse Pathogenic Parasites
Source: Front Chem. 2021 Mar 10;9:634663. doi: 10.3389/fchem.2021.634663 (PMC7987820; doi:10.3389/fchem.2021.634663)
Supplement: Supplementary file 2 [file datasheet2.pdf]

QSAR Modeling for Multi-Target Drug Discovery: Designing Simultaneous Inhibitors of Proteins in Diverse Pathogenic Parasites

Valeria V. Kleandrova[1], Luciana Scotti[2], Francisco Jaime Bezerra Mendonça Junior[3], Eugene Muratov[4], Marcus T. Scotti[2]*, and Alejandro Speck-Planche[2]*

[1]Laboratory of Fundamental and Applied Research of Quality and Technology of Food Production, Moscow State University of Food Production, Volokolamskoe shosse 11, 125080, Moscow, Russian Federation
[2]Postgraduate Program in Natural and Synthetic Bioactive Products, Federal University of Paraíba, 58051-900, João Pessoa, Brazil
[3]Laboratory of Synthesis and Drug Delivery, State University of Paraíba, João Pessoa-PB 58071-160, Brazil
[4]Laboratory for Molecular Modeling, the UNC Eshelman School of Pharmacy, University of North Carolina at Chapel Hill, Chapel Hill, NC 27599, USA

***Correspondence:** Marcus T. Scotti (mtscotti@gmail.com)
Alejandro Speck-Planche (alejspivanovich@gmail.com)

The following content describes the steps followed to generate the 3D-structure of the protein glycylpeptide N-tetradecanoyltransferase (*T. brucei brucei*) via homology modeling by the webserver SWISS-MODEL.

BIOZENTRUM University of Basel The Center for Molecular Life Sciences

SWISS-MODEL Homology Modelling Report

Model Building Report

This document lists the results for the homology modelling project "Q388H8_TRYB2 Q388H8 Glycylpeptide N-tetradecanoyltransferase" submitted to SWISS-MODEL workspace on Dec. 24, 2020, 1:58 p.m..The submitted primary amino acid sequence is given in Table T1.

If you use any results in your research, please cite the relevant publications:

- Waterhouse, A., Bertoni, M., Bienert, S., Studer, G., Tauriello, G., Gumienny, R., Heer, F.T., de Beer, T.A.P., Rempfer, C., Bordoli, L., Lepore, R., Schwede, T. SWISS-MODEL: homology modelling of protein structures and complexes. Nucleic Acids Res. 46(W1), W296-W303 (2018). doi>
- Guex, N., Peitsch, M.C., Schwede, T. Automated comparative protein structure modeling with SWISS-MODEL and Swiss-PdbViewer: A historical perspective. Electrophoresis 30, S162-S173 (2009). doi>
- Bienert, S., Waterhouse, A., de Beer, T.A.P., Tauriello, G., Studer, G., Bordoli, L., Schwede, T. The SWISS-MODEL Repository - new features and functionality. Nucleic Acids Res. 45, D313-D319 (2017). doi>
- Studer, G., Rempfer, C., Waterhouse, A.M., Gumienny, G., Haas, J., Schwede, T. QMEANDisCo - distance constraints applied on model quality estimation. Bioinformatics 36, 1765-1771 (2020). doi>
- Bertoni, M., Kiefer, F., Biasini, M., Bordoli, L., Schwede, T. Modeling protein quaternary structure of homo- and hetero-oligomers beyond binary interactions by homology. Scientific Reports 7 (2017). doi>

Results

The SWISS-MODEL template library (SMTL version 2020-12-23, PDB release 2020-12-18) was searched with BLAST (Camacho et al.) and HHBlits (Steinegger et al.) for evolutionary related structures matching the target sequence in Table T1. For details on the template search, see Materials and Methods. Overall 1070 templates were found (Table T2).

Models

The following model was built (see Materials and Methods "Model Building"):

Model #01	File	Built with	Oligo-State	Ligands	GMQE	QMEAN
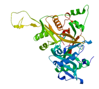	PDB	ProMod3 3.2.0	monomer	None	0.80	-1.94

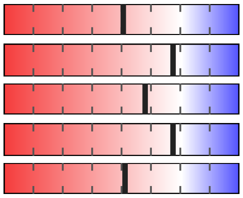

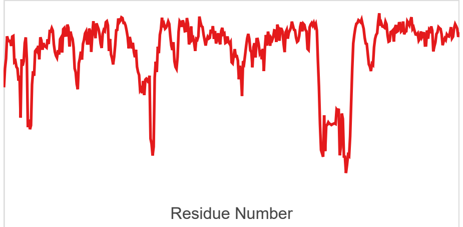

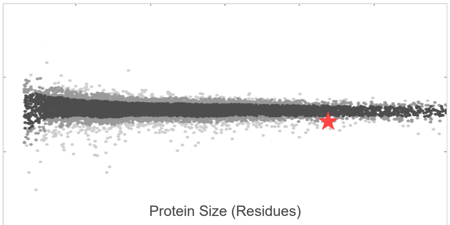

Template	Seq Identity	Oligo-state	QSQE	Found by	Method	Resolution	Seq Similarity	Range	Coverage	Description
6qd9.1.A	57.52	monomer	0.00	HHblits	X-ray	1.46Å	0.48	8 - 446	0.92	Glycylpeptide N-tetradecanoyltransferase

Excluded ligands

Ligand Name.Number	Reason for Exclusion	Description
HWT.2	Binding site not conserved.	3-[[2-[3-(dimethylamino)propyl-methyl-amino]thieno[3,2-d]pyrimidin-4-yl]-methyl-amino]propanenitrile
MG.3	Binding site not conserved.	MAGNESIUM ION

Ligand Name.Number	Reason for Exclusion	Description
MYA.1	Binding site not conserved.	TETRADECANOYL-COA

```
Target    MTDKAFTEHQFWSTQPVRQPGAPD--ADKVGFIMES-SLDAVPAEPYSLPSTFEWWSPDVANPEDLRGVHELLRDNYVED
6qd9.1.A  ---NSDAAHAFWSTQPVPQTEDETEKIVFAGPMDEPKTVADIPEEPYPIASTFEWWTPNMEAADDIHAIYELLRDNYVED

Target    SESMFRFNYSEEFLRWALMPPGYHQSWHVGVRLKSNKSVLGFVAGVPITMRLGTPKMVLEKREHGEDGGEEVINDYLEPQ
6qd9.1.A  DDSMFRFNYSEEFLQWALCPPNYIPDWHVAVRRKADKKLLAFIAGVPVTLRMGTPKYMKVKAQEKGEGEEA--AKYDEPR

Target    TICEINFLCVHKKLRQRRLGPILIKEVTRRVNLMNIWHAVYTSGTLLPTPFAKGHYFHRSLNSQKLVDVKFSGIPPHYKR
6qd9.1.A  HICEINFLCVHKQLREKRLAPILIKEATRRVNRTNVWQAVYTAGVLLPTPYASGQYFHRSLNPEKLVEIRFSGIPAQYQK

Target    FQNPVAVMERLYRLPDKTKTRGLRLMEPADVPQVTQLLLKRLASFDVAPVFNEEEVAHYFLPREGVVFSYVVESPVGPGK
6qd9.1.A  FQNPMAMLKRNYQLPSAPKNSGLREMKPSDVPQVRRILMNYLDSFDVGPVFSDAEISHYLLPRDGVVFTYVVEN------

Target    DEENAGKASKGTPTGTKCVTGGCEKVITDFFSFYSLPSTIIGNSNHSLLKVAYVYYTAATSVSITQLVNDLLIIVKLNGF
6qd9.1.A  -----------------------DKKVTDFFSFYRIPSTVIGNSNYNLLNAAYVHYYAATSIPLHQLILDLLIVAHSRGF

Target    DVCNVVDIYDNGTYLKELKFSPGDGNLYYYFYNWSYPSIPANEVGLVMV
6qd9.1.A  DVCNMVEILDNRSFVEQLKFGAGDGHLRYYFYNWAYPKIKPSQVALVML
```

Materials and Methods

Template Search

Template search with BLAST and HHBlits has been performed against the SWISS-MODEL template library (SMTL, last update: 2020-12-23, last included PDB release: 2020-12-18).

The target sequence was searched with BLAST against the primary amino acid sequence contained in the SMTL. A total of 52 templates were found.

An initial HHblits profile has been built using the procedure outlined in (Steinegger et al.), followed by 1 iteration of HHblits against Uniclust30 (Mirdita, von den Driesch et al.). The obtained profile has then be searched against all profiles of the SMTL. A total of 1020 templates were found.

Template Selection

For each identified template, the template's quality has been predicted from features of the target-template alignment. The templates with the highest quality have then been selected for model building.

Model Building

Models are built based on the target-template alignment using ProMod3. Coordinates which are conserved between the target and the template are copied from the template to the model. Insertions and deletions are remodelled using a fragment library. Side chains are then rebuilt. Finally, the geometry of the resulting model is regularized by using a force field. In case loop modelling with ProMod3 fails, an alternative model is built with PROMOD-II (Guex et al.).

Model Quality Estimation

The global and per-residue model quality has been assessed using the QMEAN scoring function (Studer et al.).

Ligand Modelling

Ligands present in the template structure are transferred by homology to the model when the following criteria are met: (a) The ligands are annotated as biologically relevant in the template library, (b) the ligand is in contact with the model, (c) the ligand is not clashing with the protein, (d) the residues in contact with the ligand are conserved between the target and the template. If any of these four criteria is not satisfied, a certain ligand will not be included in the model. The model summary includes information on why and which ligand has not been included.

Oligomeric State Conservation

The quaternary structure annotation of the template is used to model the target sequence in its oligomeric form. The method (Bertoni et al.) is based on a supervised machine learning algorithm, Support Vector Machines (SVM), which combines interface conservation, structural clustering, and other template features to provide a quaternary structure quality estimate (QSQE). The QSQE score is a number between 0 and 1, reflecting the expected accuracy of the interchain contacts for a model built based a given alignment and template. Higher numbers indicate higher reliability. This complements the GMQE score which estimates the accuracy of the tertiary structure of the resulting model.

References

- **BLAST**
  Camacho, C., Coulouris, G., Avagyan, V., Ma, N., Papadopoulos, J., Bealer, K., Madden, T.L. BLAST+: architecture and applications. BMC Bioinformatics 10, 421-430 (2009). doi>
- **HHblits**
  Steinegger, M., Meier, M., Mirdita, M., Vöhringer, H., Haunsberger, S. J., Söding, J. HH-suite3 for fast remote homology detection and deep protein annotation. BMC Bioinformatics 20, 473 (2019). doi>
- **Uniclust30**
  Mirdita, M., von den Driesch, L., Galiez, C., Martin, M.J., Söding, J., Steinegger, M. Uniclust databases of clustered and deeply annotated protein sequences and alignments. Nucleic Acids Research 45, D170–D176 (2016). doi>

Table T1:

Primary amino acid sequence for which templates were searched and models were built.

```
MTDKAFTEHQFWSTQPVRQPGAPDADKVGFIMESSLDAVPAEPYSLPSTFEWWSPDVANPEDLRGVHELLRDNYVEDSESMFRFNYSEEFLRWALMPPGY
HQSWHVGVRLKSNKSVLGFVAGVPITMRLGTPKMVLEKREHGEDGGEEVINDYLEPQTICEINFLCVHKKLRQRRLGPILIKEVTRRVNLMNIWHAVYTS
GTLLPTPFAKGHYFHRSLNSQKLVDVKFSGIPPHYKRFQNPVAVMERLYRLPDKTKTRGLRLMEPADVPQVTQLLLKRLASFDVAPVFNEEEVAHYFLPR
EGVVFSYVVESPVGPGKDEENAGKASKGTPTGTKCVTGGCEKVITDFFSFYSLPSTIIGNSNHSLLKVAYVYYTAATSVSITQLVNDLLIIVKLNGFDVC
NVVDIYDNGTYLKELKFSPGDGNLYYYFYNWSYPSIPANEVGLVMV
```

Table T2:

Template	Seq Identity	Oligo-state	QSQE	Found by	Method	Resolution	Seq Similarity	Coverage	Description
6qd9.1.A	57.52	monomer	-	HHblits	X-ray	1.46Å	0.48	0.92	Glycylpeptide N-tetradecanoyltransferase
4ucn.1.A	57.38	monomer	-	HHblits	X-ray	1.80Å	0.48	0.93	GLYCYLPEPTIDE N-TETRADECANOYLTRANSFERASE
2wsa.1.A	57.63	monomer	-	HHblits	X-ray	1.60Å	0.48	0.93	GLYCYLPEPTIDE N-TETRADECANOYLTRANSFERASE
5g20.1.A	58.09	monomer	-	HHblits	X-ray	1.52Å	0.48	0.91	GLYCYLPEPTIDE N-TETRADECANOYLTRANSFERASE
6gnu.1.A	57.38	monomer	-	HHblits	X-ray	1.54Å	0.48	0.93	Glycylpeptide N-tetradecanoyltransferase
4ucn.1.A	59.75	monomer	-	BLAST	X-ray	1.80Å	0.50	0.91	GLYCYLPEPTIDE N-TETRADECANOYLTRANSFERASE
4cgm.1.A	58.09	monomer	-	HHblits	X-ray	1.70Å	0.48	0.91	GLYCYLPEPTIDE N-TETRADECANOYLTRANSFERASE
6qd9.1.A	59.75	monomer	-	BLAST	X-ray	1.46Å	0.50	0.91	Glycylpeptide N-tetradecanoyltransferase
6omk.1.A	57.77	monomer	-	HHblits	X-ray	2.10Å	0.48	0.92	Glycylpeptide N-tetradecanoyltransferase
5g20.1.A	59.75	monomer	-	BLAST	X-ray	1.52Å	0.50	0.91	GLYCYLPEPTIDE N-TETRADECANOYLTRANSFERASE
6gnu.1.A	59.75	monomer	-	BLAST	X-ray	1.54Å	0.50	0.91	Glycylpeptide N-tetradecanoyltransferase
2wsa.1.A	60.00	monomer	-	BLAST	X-ray	1.60Å	0.50	0.91	GLYCYLPEPTIDE N-TETRADECANOYLTRANSFERASE
4cgm.1.A	59.75	monomer	-	BLAST	X-ray	1.70Å	0.50	0.91	GLYCYLPEPTIDE N-TETRADECANOYLTRANSFERASE
6omk.1.A	60.00	monomer	-	BLAST	X-ray	2.10Å	0.50	0.91	Glycylpeptide N-tetradecanoyltransferase

Template	Seq Identity	Oligo-state	QSQE	Found by	Method	Resolution	Seq Similarity	Coverage	Description
6gnv.1.A	58.77	monomer	-	BLAST	X-ray	2.20Å	0.49	0.91	Glycylpeptide N-tetradecanoyltransferase
2wuu.1.A	57.52	monomer	-	HHblits	X-ray	1.42Å	0.48	0.92	N-MYRISTOYLTRANSFERASE
6gnt.1.A	59.75	monomer	-	BLAST	X-ray	1.60Å	0.50	0.91	Glycylpeptide N-tetradecanoyltransferase
2wuu.1.A	58.62	monomer	-	BLAST	X-ray	1.42Å	0.49	0.91	N-MYRISTOYLTRANSFERASE
4c2y.1.A	45.60	monomer	-	HHblits	X-ray	1.64Å	0.43	0.87	GLYCYLPEPTIDE N-TETRADECANOYLTRANSFERASE 1
6f56.1.A	45.85	homo-tetramer	0.42	HHblits	X-ray	1.94Å	0.43	0.87	Glycylpeptide N-tetradecanoyltransferase 1
6f56.1.B	45.85	homo-tetramer	0.42	HHblits	X-ray	1.94Å	0.43	0.87	Glycylpeptide N-tetradecanoyltransferase 1
4c2x.1.A	49.48	monomer	-	HHblits	X-ray	2.33Å	0.44	0.87	GLYCYLPEPTIDE N-TETRADECANOYLTRANSFERASE 2
6f56.1.D	45.85	homo-tetramer	0.42	HHblits	X-ray	1.94Å	0.43	0.87	Glycylpeptide N-tetradecanoyltransferase 1
4c2z.2.A	45.60	monomer	-	HHblits	X-ray	2.08Å	0.43	0.87	GLYCYLPEPTIDE N-TETRADECANOYLTRANSFERASE 1
4c2y.1.A	47.03	monomer	-	BLAST	X-ray	1.64Å	0.44	0.87	GLYCYLPEPTIDE N-TETRADECANOYLTRANSFERASE 1
4c2x.1.A	50.78	monomer	-	BLAST	X-ray	2.33Å	0.45	0.87	GLYCYLPEPTIDE N-TETRADECANOYLTRANSFERASE 2
6f56.1.A	47.16	homo-tetramer	0.41	BLAST	X-ray	1.94Å	0.44	0.87	Glycylpeptide N-tetradecanoyltransferase 1
5npq.2.A	45.48	monomer	-	HHblits	X-ray	2.37Å	0.43	0.87	Glycylpeptide N-tetradecanoyltransferase 1
6f56.1.B	47.16	homo-tetramer	0.41	BLAST	X-ray	1.94Å	0.44	0.87	Glycylpeptide N-tetradecanoyltransferase 1
6pav.1.A	45.62	homo-dimer	0.39	HHblits	X-ray	2.52Å	0.43	0.87	Glycylpeptide N-tetradecanoyltransferase 1
6f56.1.D	47.16	homo-tetramer	0.41	BLAST	X-ray	1.94Å	0.44	0.87	Glycylpeptide N-tetradecanoyltransferase 1
5npq.2.A	47.03	monomer	-	BLAST	X-ray	2.37Å	0.44	0.87	Glycylpeptide N-tetradecanoyltransferase 1
4c2z.2.A	47.03	monomer	-	BLAST	X-ray	2.08Å	0.44	0.87	GLYCYLPEPTIDE N-TETRADECANOYLTRANSFERASE 1
5o9s.1.A	45.60	monomer	-	HHblits	X-ray	2.70Å	0.43	0.87	Glycylpeptide N-tetradecanoyltransferase 1
6pav.1.A	47.03	homo-dimer	0.38	BLAST	X-ray	2.52Å	0.44	0.87	Glycylpeptide N-tetradecanoyltransferase 1
6pau.1.A	49.74	monomer	-	HHblits	X-ray	1.93Å	0.44	0.86	Glycylpeptide N-tetradecanoyltransferase 2
5o9s.1.A	47.03	monomer	-	BLAST	X-ray	2.70Å	0.44	0.87	Glycylpeptide N-tetradecanoyltransferase 1
6pau.1.A	51.45	monomer	-	BLAST	X-ray	1.93Å	0.45	0.85	Glycylpeptide N-tetradecanoyltransferase 2
5v0w.1.A	44.47	monomer	-	BLAST	X-ray	1.80Å	0.43	0.85	Glycylpeptide N-tetradecanoyltransferase
4a95.1.A	44.47	monomer	-	BLAST	X-ray	1.55Å	0.43	0.85	GLYCYLPEPTIDE N-TETRADECANOYLTRANSFERASE

Template	Seq Identity	Oligo-state	QSQE	Found by	Method	Resolution	Seq Similarity	Coverage	Description
2ynd.1.A	44.47	monomer	-	BLAST	X-ray	1.89Å	0.43	0.85	GLYCYLPEPTIDE N-TETRADECANOYLTRANSFERASE
4b10.1.A	44.47	monomer	-	BLAST	X-ray	1.56Å	0.43	0.85	GLYCYLPEPTIDE N-TETRADECANOYLTRANSFERASE
6nxg.3.A	44.47	monomer	-	BLAST	X-ray	1.60Å	0.43	0.85	Glycylpeptide N-tetradecanoyltransferase
1iic.2.A	42.97	monomer	-	HHblits	X-ray	2.20Å	0.42	0.86	PEPTIDE N-myristoyltransferase
1iic.1.A	42.97	monomer	-	HHblits	X-ray	2.20Å	0.42	0.86	PEPTIDE N-myristoyltransferase
2ync.3.A	44.21	monomer	-	BLAST	X-ray	1.75Å	0.43	0.85	GLYCYLPEPTIDE N-TETRADECANOYLTRANSFERASE
2p6e.1.A	43.15	monomer	-	HHblits	X-ray	2.90Å	0.42	0.87	Glycylpeptide N-tetradecanoyltransferase
5g22.3.A	44.47	monomer	-	BLAST	X-ray	2.32Å	0.43	0.85	GLYCYLPEPTIDE N-TETRADECANOYLTRANSFERASE
2p6f.6.A	43.15	monomer	-	HHblits	X-ray	3.10Å	0.42	0.87	Glycylpeptide N-tetradecanoyltransferase
6b1l.2.A	44.47	monomer	-	BLAST	X-ray	2.30Å	0.43	0.85	Glycylpeptide N-tetradecanoyltransferase

The table above shows the top 50 filtered templates. A further 550 templates were found which were considered to be less suitable for modelling than the filtered list.
1b6b.1.A, 1b6b.2.A, 1b87.1.A, 1bo4.1.A, 1cjw.1.A, 1cm0.1.A, 1cm0.2.A, 1ghe.1.A, 1i12.1.A, 1i12.2.B, 1i1d.1.A, 1i1d.1.B, 1i21.1.A, 1i21.3.A, 1ib1.1.C, 1iic.1.A, 1iic.2.A, 1iyk.1.A, 1iyk.2.A, 1iyl.1.A, 1iyl.2.A, 1iyl.3.A, 1iyl.4.A, 1kux.1.A, 1l0c.1.A, 1m1d.1.A, 1m1d.2.A, 1m44.1.A, 1m4g.1.A, 1m4i.1.B, 1mk4.1.A, 1mk4.1.B, 1n71.1.A, 1nmt.1.A, 1on0.1.A, 1on0.1.C, 1on0.1.D, 1p0h.1.A, 1pu9.1.A, 1pua.1.A, 1q2c.1.A, 1q2y.1.A, 1qsm.1.A, 1qso.1.A, 1qso.1.B, 1qso.1.C, 1qso.1.D, 1qsr.1.A, 1qst.1.A, 1r57.1.A, 1rxt.1.A, 1s3z.1.A, 1s3z.1.B, 1s5k.1.A, 1s60.1.A, 1sqh.1.A, 1suy.1.C, 1sv1.1.C, 1tiq.1.A, 1tiq.2.A, 1u6m.1.A, 1u6m.3.A, 1ufh.1.A, 1ufh.1.B, 1vkc.1.A, 1wk4.1.A, 1wk4.2.A, 1wk4.3.A, 1wwz.1.A, 1wwz.1.B, 1xeb.1.A, 1xeb.4.A, 1xeb.5.A, 1y7r.1.A, 1y7r.1.B, 1y9k.1.A, 1y9k.2.A, 1y9w.1.A, 1y9w.1.B, 1yr0.1.A, 1yre.1.A, 1yre.1.B, 1yx0.1.A, 1z4e.1.A, 1z4r.1.A, 2a4n.1.A, 2a4n.1.B, 2ae6.1.A, 2ae6.1.C, 2aj6.1.A, 2atr.1.A, 2b3u.1.B, 2b4b.1.A, 2b4b.1.B, 2b4d.1.A, 2b4d.1.B, 2b58.1.A, 2b5g.1.A, 2b5g.1.B, 2c27.1.A, 2cnm.1.A, 2cnt.1.A, 2cy2.1.A, 2dxq.1.A, 2dxq.1.B, 2eui.1.A, 2eui.1.B, 2eui.2.A, 2evn.1.A, 2f5i.1.A, 2f5i.1.B, 2fck.1.A, 2fe7.1.A, 2fia.1.A, 2fiw.1.A, 2fxf.1.A, 2fxf.1.B, 2g3a.1.A, 2gan.1.A, 2gan.1.B, 2h5m.1.A, 2huz.1.A, 2huz.1.B, 2hv2.1.A, 2i00.1.A, 2i79.1.A, 2i79.2.A, 2jdc.1.A, 2jev.1.A, 2jlm.1.A, 2k5t.1.A, 2kcw.1.A, 2nmt.1.A, 2oh1.1.A, 2oh1.2.A, 2ozg.1.A, 2ozh.1.A, 2p6e.1.A, 2p6f.6.A, 2pc1.1.A, 2pdo.1.A, 2pdo.1.B, 2pdo.2.B, 2pdo.4.A, 2pdo.4.B, 2pr1.1.A, 2pr1.1.B, 2prb.1.A, 2psw.1.A, 2q0y.1.A, 2q44.1.A, 2q4v.1.A, 2q4v.1.B, 2q4y.1.A, 2qec.1.A, 2qml.1.A, 2r1i.1.A, 2r7h.1.A, 2r7h.1.B, 2ree.1.A, 2wpw.1.A, 2wpx.1.A, 2wuu.1.A, 2x7b.1.A, 2ync.3.A, 2ynd.1.A, 2zpa.1.A, 2zpa.2.A, 3bj7.1.A, 3bj7.1.B, 3bj7.2.A, 3bj8.1.A, 3bj8.1.B, 3bj8.2.A, 3bj8.2.B, 3bln.1.A, 3c26.1.A, 3cxp.1.A, 3cxq.1.B, 3d2m.1.A, 3d2p.1.A, 3d3s.1.A, 3d3s.1.B, 3d3s.2.A, 3d3s.2.B, 3d8p.1.A, 3dr6.1.A, 3dr6.2.A, 3dr8.1.A, 3dr8.1.B, 3dsb.1.A, 3e0k.1.A, 3ec4.1.A, 3efa.1.A, 3eo4.1.A, 3eo4.2.B, 3exn.1.A, 3ey5.1.A, 3f0a.1.A, 3f8k.1.A, 3fb3.1.A, 3fb3.1.B, 3fix.1.A, 3fix.2.A, 3fnc.1.A, 3fnc.1.B, 3frm.1.A, 3frm.1.B, 3frm.2.B, 3frm.3.A, 3frm.4.A, 3fyn.1.A, 3g3s.1.A, 3gy9.1.A, 3i3g.1.A, 3i3g.1.B, 3i9s.1.A, 3iu1.2.A, 3iwg.1.A, 3jtk.1.A, 3jvn.1.A, 3k9u.1.A, 3kkw.1.A, 3ld2.1.A, 3ld2.3.A, 3lod.1.A, 3mgd.1.A, 3n7z.1.A, 3n7z.1.B, 3ne7.1.A, 3owc.1.A, 3p2h.1.A, 3pzj.1.A, 3pzj.1.B, 3qb8.1.A, 3r1k.1.A, 3ryo.1.A, 3s6f.1.A, 3shp.1.A, 3shp.1.B, 3sxn.1.A, 3sxo.1.A, 3t90.1.A, 3t9y.1.A, 3tcv.1.A, 3te4.1.A, 3tfy.1.A, 3tt2.1.A, 3v8i.1.A, 3w6s.1.A, 3w6x.6.B, 3w91.1.A, 3zj0.1.A, 4a95.1.A, 4ag7.1.A, 4ag7.1.B, 4ag9.1.B, 4b10.1.A, 4bmh.1.A, 4c2x.1.A, 4c2y.1.A, 4c2z.2.A, 4cav.1.A, 4caw.1.A, 4cgm.1.A, 4cry.1.B, 4e0a.1.A, 4e0a.1.B, 4e2a.1.A, 4e8o.1.A, 4evy.1.A, 4evy.1.B, 4f0y.1.A, 4f0y.1.B, 4f6a.1.A, 4fd4.1.A, 4fd4.2.A, 4fd6.1.A, 4fd7.1.A, 4fd7.3.A, 4fd7.4.A, 4h89.1.A, 4hnw.1.B, 4hnx.1.B, 4hny.1.B, 4i49.1.E, 4ius.1.A, 4jd6.1.A, 4jwp.1.B, 4jxr.1.A, 4kvo.3.B, 4kvx.1.A, 4l89.1.A, 4lx9.1.A, 4m3s.1.A, 4m85.1.A, 4m85.2.A, 4mxe.1.A, 4mxe.1.B, 4my0.1.C, 4my3.1.A, 4my3.1.B, 4my3.1.D, 4oae.1.A, 4pv6.1.A, 4pv6.10.A, 4pv6.11.A, 4pv6.12.A, 4pv6.13.A, 4pv6.14.A, 4pv6.15.A, 4pv6.16.A, 4pv6.2.A, 4pv6.4.A, 4pv6.5.A, 4pv6.6.A, 4pv6.7.A, 4pv6.8.A, 4pv6.9.A, 4qb9.1.B, 4qb9.1.C, 4qb9.1.D, 4qb9.1.E, 4qbj.1.A, 4qus.1.A, 4qus.1.B, 4qvt.1.A, 4r3k.1.A, 4ri1.1.A, 4rs2.1.A, 4rs2.2.A, 4u9v.1.A, 4u9w.1.A, 4ua3.1.A, 4ua3.2.A, 4ubr.1.A, 4uwi.1.A, 4xpd.1.B, 4xpd.1.C, 4y49.1.B, 4y49.1.C, 4yfj.1.A, 4yfj.1.B, 4zbg.1.A, 4zm6.1.A, 5c88.1.A, 5dwn.1.A, 5e96.1.B, 5ebv.1.F, 5f46.1.A, 5f46.1.B, 5f47.2.D, 5f48.1.A, 5f49.2.A, 5fvj.1.A, 5g20.1.A, 5g22.3.A, 5gcn.1.A, 5gi6.1.A, 5gif.1.A, 5gii.1.A, 5hgz.1.A, 5hh0.1.A, 5hh1.1.A, 5hmn.1.A, 5hmn.1.B, 5hmn.2.A, 5hmn.2.B, 5i0c.1.A, 5ib0.1.A, 5icv.1.A, 5icw.1.A, 5icw.2.A, 5isv.1.A, 5jph.1.A, 5jq4.1.A, 5jq4.2.A, 5jtf.1.B, 5k04.1.B, 5k18.1.B, 5k9n.1.A, 5k9n.2.A, 5kf2.1.A, 5kga.1.A, 5kgh.1.A, 5kta.1.A, 5ktc.1.A, 5n1u.1.A, 5n1u.2.A, 5n1w.1.A, 5n22.1.A, 5n22.2.A, 5n22.4.A, 5nnp.1.B, 5nnr.1.B, 5nnr.2.B, 5npq.2.A, 5o7o.1.A, 5o9s.1.A, 5t53.1.A, 5t7d.1.A, 5t7e.2.A, 5trl.1.P, 5trl.1.S, 5trl.1.W, 5trm.1.A, 5trm.1.B, 5trm.1.C, 5trm.1.E, 5trm.1.F, 5trm.1.H, 5trm.1.I, 5trm.1.J, 5trm.1.K, 5trm.1.L, 5trm.1.M, 5trm.1.O, 5trm.1.P, 5trm.1.Q, 5trm.1.S, 5trm.1.T, 5trm.1.W, 5trm.1.X, 5u08.1.A, 5u08.1.B, 5u08.2.A, 5us1.1.A, 5us1.1.B, 5us1.2.A, 5us1.2.B, 5us1.3.B, 5us1.4.A, 5us1.4.B, 5us1.5.B, 5us1.6.B, 5v0w.1.A, 5vdb.1.A, 5w8a.1.A, 5w8c.1.A, 5wjd.1.A, 5wje.1.A, 5wph.1.A, 5wph.1.B, 5wph.3.B, 5xun.1.A, 5yge.1.A, 5yo2.1.A, 5z6n.1.A, 5z6n.1.B, 5zgn.1.C, 6ag4.1.A, 6ag5.1.A, 6ajm.1.A, 6ajm.1.B, 6ajn.1.A, 6ajn.1.B, 6ao7.1.A, 6b1l.2.A, 6bvc.1.A, 6c95.1.B, 6c9m.1.B, 6cw2.2.A, 6cw3.1.C, 6cw3.2.C, 6erd.1.A, 6erd.1.B, 6f56.1.A, 6f56.1.B, 6f56.1.D, 6fz2.1.A, 6fz5.1.A, 6g96.1.A, 6g96.1.B, 6gnt.1.A, 6gnv.1.A, 6gtp.1.A, 6gtq.1.A, 6gtq.2.A, 6gts.1.A, 6hd7.1.m, 6hd7.1.n, 6iuf.1.A, 6k5m.1.A, 6k80.1.A, 6m7g.4.A, 6may.1.A, 6mfc.1.A, 6mfd.2.A, 6nas.1.B, 6nxg.3.A, 6o07.1.A, 6o07.1.C, 6omk.1.A, 6pau.1.A, 6pav.1.A, 6ppl.1.A,

6ppl.1.C, 6pw9.1.A, 6pw9.1.C, 6rft.1.A, 6rfx.1.A, 6sjy.1.A, 6sjy.1.B, 6sjy.1.C, 6sk1.1.A, 6sk3.1.A, 6skj.1.A, 6sl8.1.A, 6slk.1.A, 6slk.1.B, 6slk.1.E, 6sll.1.A, 6sll.1.B, 6sp0.1.A, 6tdg.1.B, 6tdh.1.B, 6tgx.1.A, 6tgx.2.A, 6th0.1.A, 6th0.2.A, 6v3t.1.A, 6vp9.1.A, 6wf3.1.A, 6wf5.1.A, 6wfn.1.A, 6wn0.1.A, 6wns.1.A, 6yca.1.A, 6yga.1.A, 6ygd.1.A, 6yug.1.A, 6yug.1.B, 6zmp.1.A, 7bxz.1.A, 7bxz.1.B, 7chd.1.A, 7chd.3.A, 7chd.3.B, 7chd.4.A, 7chd.5.A, 7chd.5.B, 7kwq.1.A, 7kwq.1.B, 7kwq.1.C, 7kx2.1.A, 7kx2.1.B, 7kx2.1.C, 7kx3.1.A, 7kx3.1.B, 7kx3.1.C
