# Supplementary material for: QSAR Modeling for Multi-Target Drug Discovery: Designing Simultaneous Inhibitors of Proteins in Diverse Pathogenic Parasites
Source: Front Chem. 2021 Mar 10;9:634663. doi: 10.3389/fchem.2021.634663 (PMC7987820; doi:10.3389/fchem.2021.634663)
Supplement: Supplementary file 3 [file datasheet3.pdf]

QSAR Modeling for Multi-Target Drug Discovery: Designing Simultaneous Inhibitors of Proteins in Diverse Pathogenic Parasites

Valeria V. Kleandrova[1], Luciana Scotti[2], Francisco Jaime Bezerra Mendonça Junior[3], Eugene Muratov[4], Marcus T. Scotti[2]*, and Alejandro Speck-Planche[2]*

[1]Laboratory of Fundamental and Applied Research of Quality and Technology of Food Production, Moscow State University of Food Production, Volokolamskoe shosse 11, 125080, Moscow, Russian Federation
[2]Postgraduate Program in Natural and Synthetic Bioactive Products, Federal University of Paraíba, 58051-900, João Pessoa, Brazil
[3]Laboratory of Synthesis and Drug Delivery, State University of Paraíba, João Pessoa-PB 58071-160, Brazil
[4]Laboratory for Molecular Modeling, the UNC Eshelman School of Pharmacy, University of North Carolina at Chapel Hill, Chapel Hill, NC 27599, USA

***Correspondence:** Marcus T. Scotti (mtscotti@gmail.com)
Alejandro Speck-Planche (alejspivanovich@gmail.com)

Table 1.SI7 Assessment of the druglikeness and synthetic accessibility of the designed molecules.

ID[a]	HBD	HBA	MW (Da)	MlogP	AlogP	nAT	MR (cm^3/mol)	RBN	PSA ($Å^2$)	SAS
MTIPP-001	2	6	458.38	3.674	4.881	52	120.599	3	79.26	3.48
MTIPP-002	2	6	466.78	3.463	4.845	48	117.636	3	79.26	3.31
MTIPP-003	3	6	434.75	1.926	3.491	40	102.314	4	110.53	3.23
MTIPP-004	3	6	434.75	1.926	3.491	40	102.314	4	110.53	3.24

[a]The following symbols are: **HBD** – number of hydrogen bond donors; **HBA** – number of hydrogen bond acceptors; **MW** – molecular weight; **MlogP** – logarithm of the partition coefficient (octanol/water) estimated according to the Moriguchi's approach; **AlogP** – logarithm of the partition coefficient (octanol/water) estimated according to the Ghose-Crippen's approach; **nAT** – number of atoms; **MR** – molar refractivity; **RBN** – number of rotatable bonds; **PSA** – polar surface area considering nitrogen, oxygen, sulfur, and phosphorus; **SAS** – synthetic accessibility score.
